# Two-Level Protein Methylation Prediction using structure model-based features

**DOI:** 10.1038/s41598-020-62883-2

**Published:** 2020-04-07

**Authors:** Wei Zheng, Qiqige Wuyun, Micah Cheng, Gang Hu, Yanping Zhang

**Affiliations:** 1 Department of Computational Medicine and Bioinformatics, University of Michigan, Ann Arbor, MI 48109 USA; 2 Computer Science and Engineering Department, Michigan State University, East Lansing, MI 48823 USA; 3 School of Mathematical Sciences and LPMC, Nankai University, Tianjin, 300071 PR China; 4 Department of Electrical Engineering and Computer Science, University of Michigan, Ann Arbor, MI 48109 USA; 5 Department of Mathematics, School of Mathematics and Physics, Hebei University of Engineering, Handan, 056038 PR China

**Keywords:** Bioinformatics, Protein function predictions

## Abstract

Protein methylation plays a vital role in cell processing. Many novel methods try to predict methylation sites from protein sequence by sequence information or predicted structural information, but none of them use protein tertiary structure information in prediction. In particular, most of them do not build models for predicting methylation types (mono-, di-, tri-methylation). To address these problems, we propose a novel method, Met-predictor, to predict methylation sites and methylation types using a support vector machine-based network. Met-predictor combines a variety of sequence-based features that are derived from protein sequences with structure model-based features, which are geometric information extracted from predicted protein tertiary structure models, and are firstly used in methylation prediction. Met-predictor was tested on two independent test sets, where the addition of structure model-based features improved AUC from 0.611 and 0.520 to 0.655 and 0.566 for lysine and from 0.723 and 0.640 to 0.734 and 0.643 for arginine. When compared with other state-of-the-art methods, Met-predictor had 13.1% (3.9%) and 8.5% (16.4%) higher accuracy than the best of other methods for methyllysine and methylarginine prediction on the independent test set I (II). Furthermore, Met-predictor also attains excellent performance for predicting methylation types.

## Introduction

**Figure 1:**
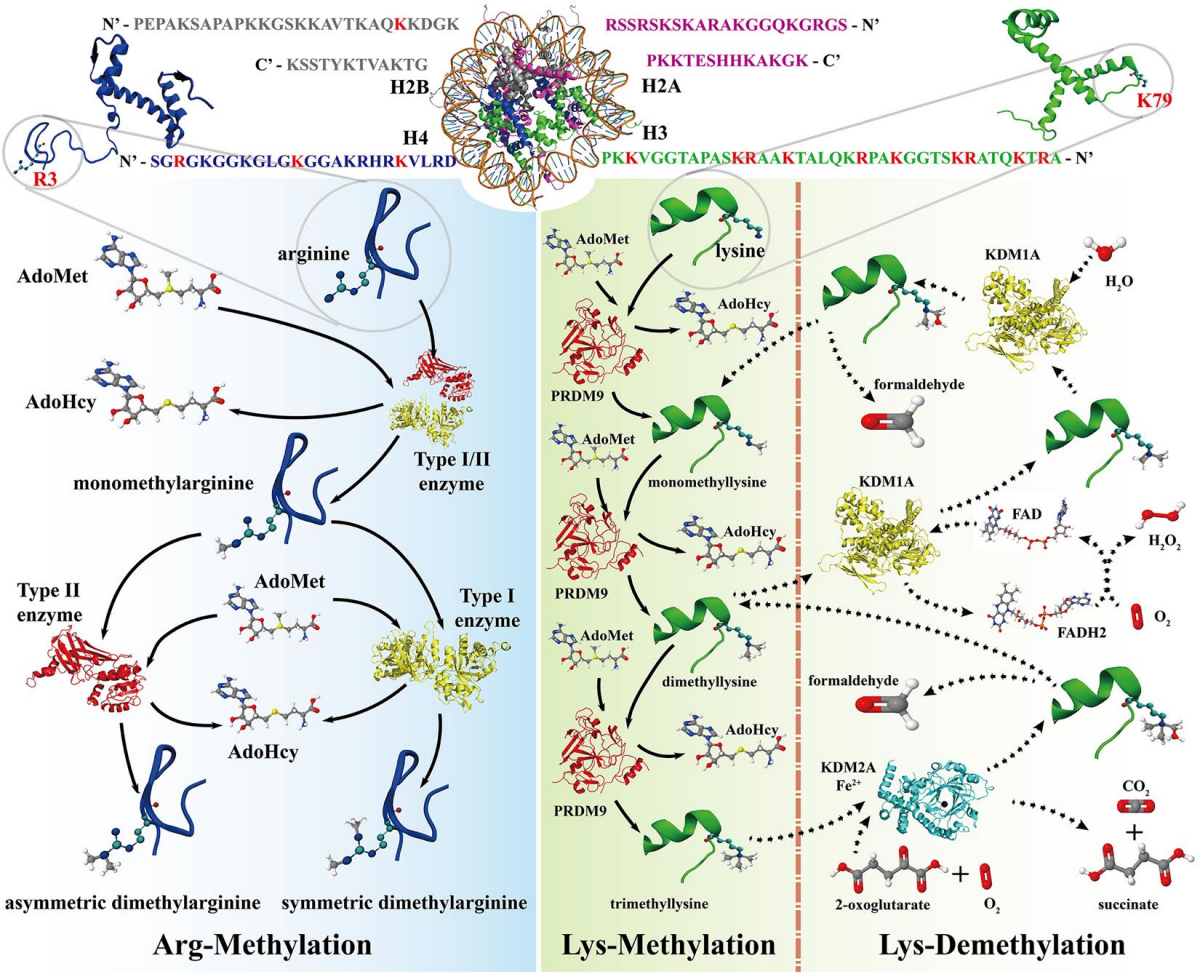
The methylation and demethylation processes for arginine and lysine. The histone on the top of the picture (PDB: 1KX5) includes four subunits: H2A, H2B, H3, and H4. There are many methylarginines and methyllysines on the H3 and H4 subunits (red letters). The methylation of arginine is shown on the left. Arginine methylation has three types: mono-methylation, symmetric di-methylation, and asymmetric di-methylation, which can be catalysed by Type I/II protein arginine methyltransferases (PRMTs). The methylation and demethylation of lysine are shown on the right panel, which also includes three types: mono-methylation, di-methylation, and tri-methylation. The lysine methylation can be catalysed by histone lysine methyltransferases (HKMTs or PRDMs), whereas the demethylation of lysine can be catalysed by lysine-specific histone demethylases (KDM1A and KDM2A).

Protein methylation is one of the most important post-translational modifications^1^, which generally occurs at the lysine or arginine residues of a protein. Arginine methylation has two types: mono-methylation, which means the arginine is methylated once, and di-methylation, which can be symmetric or asymmetric, where the arginine residue is methylated twice (Fig. 1). The process of arginine methylation can be catalysed by protein arginine methyltransferases (PRMTs). The arginine methylation can be detected in transcription regulation, RNA processing, signal transduction, DNA repair, genome stability, and some cancers^2^. Similarly, lysine methylation mainly occurs on histones, which involves the addition of one to three methyl groups to the ε-Nitrogen atom on the lysine residue, catalysed by histone lysine methyltransferases (HKMTs or PRDMs). In other words, the lysine can be methylated once, twice, and three times (Fig. 1), corresponding to the three types of lysine methylation: mono-methylation, di-methylation, and tri-methylation. The lysine methylation has been widely studied in H3 and H4 histone proteins, which play a vital role in various protein processes, such as heterochromatin compaction, X-Chromosome inactivation, and transcriptional silencing or activation^3,4^. Recent evidence has found that HKMTs can also modify the function of some non-histone proteins^5,6^.

Since the arginine methylation and lysine methylation play important roles in gene regulation, many human diseases are related to them, such as cancer, coronary heart disease, multiple sclerosis, rheumatoid arthritis, and neurodegenerative disorders^7^. Thus, understanding the regulatory mechanism of methylation is essential for disease treatment. The first and fundamental step will be identifying protein methylation sites. Although experimental methods can be used to confirm the protein methylation sites, these experimental methods are often time consuming and expensive. As the number of protein sequences explodes, a large variety of alternative computational methods are required for the accurate identification of the potential protein methylation sites. Currently, various computational methods have been employed to predict methylation sites from protein sequences. Those methods can be categorized as two groups, homologous detecting-based method and machine learning-based method. The idea of homologous detecting-based method^8^ is to assign the arginine or lysine residue of the query sequence as methylation or non-methylation site based on the annotation of the aligned residue of the homologous sequence detected by BLAST^9^ which searches homologous sequences from a database of protein sequences with known methylation sites. Most of the machine learning-based methods^10–15^ make use of the support vector machine (SVM) classifier to build models, while iPTM-mLys^16^ and MePred-RF^17^ utilize the random forest (RF). The features employed in these machine learning-based methods are as diverse as: disorder^18,19^, orthogonal binary coding scheme^10^, bi-profile Bayes feature^20^, solvent accessible surface area^11^, secondary structure^11^, position-specific profiles^21^, enhanced feature encoding scheme^22^, composition of K-spaced amino acid pairs^23^, pseudo amino acid composition^21^, and K-gap amino acid pairs encoding scheme^15^. For instance, MeMo^10^ is a web server for the protein methylation prediction implemented in SVMs. MASA^11^ combines the SVM with the sequence and structural characteristics of proteins to identify methylation sites on lysine, arginine, glutamate, and asparagine. PLMLA^12^ incorporates protein grouped weight and position weight amino acid composition, secondary structure and amino acid physicochemical properties to predict methylation and acetylation of lysine residues based on an SVM classifier. PMeS^13^ is developed for the prediction of protein methylation sites based on an enhanced feature encoding scheme and SVM. MethK^14^ is constructed using SVM with amino acid composition and accessible surface area to identify lysine-methylated sites on both histones and non-histone proteins. PSSMe^24^ employs the information gain feature optimization method to identify species-specific methylation sites. iLM_2L^15^ utilizes the composition of k-spaced amino acid pairs feature coding scheme and the SVM classifier to predict lysine methylation sites and their methylation degrees. More recently, GPS-MSP^25^ is built for the prediction of different types of methyllysine and methylarginine residues in proteins using the Group-based Prediction System (GPS) algorithm.

Although these methods have their own advantages, there are still some limitations that need to be addressed. First, the datasets used in model training did not include enough updated and non-redundant methylation sites. Second, most methods only built models for methylation site prediction but did not take the methylation types into account.

**Figure 2:**
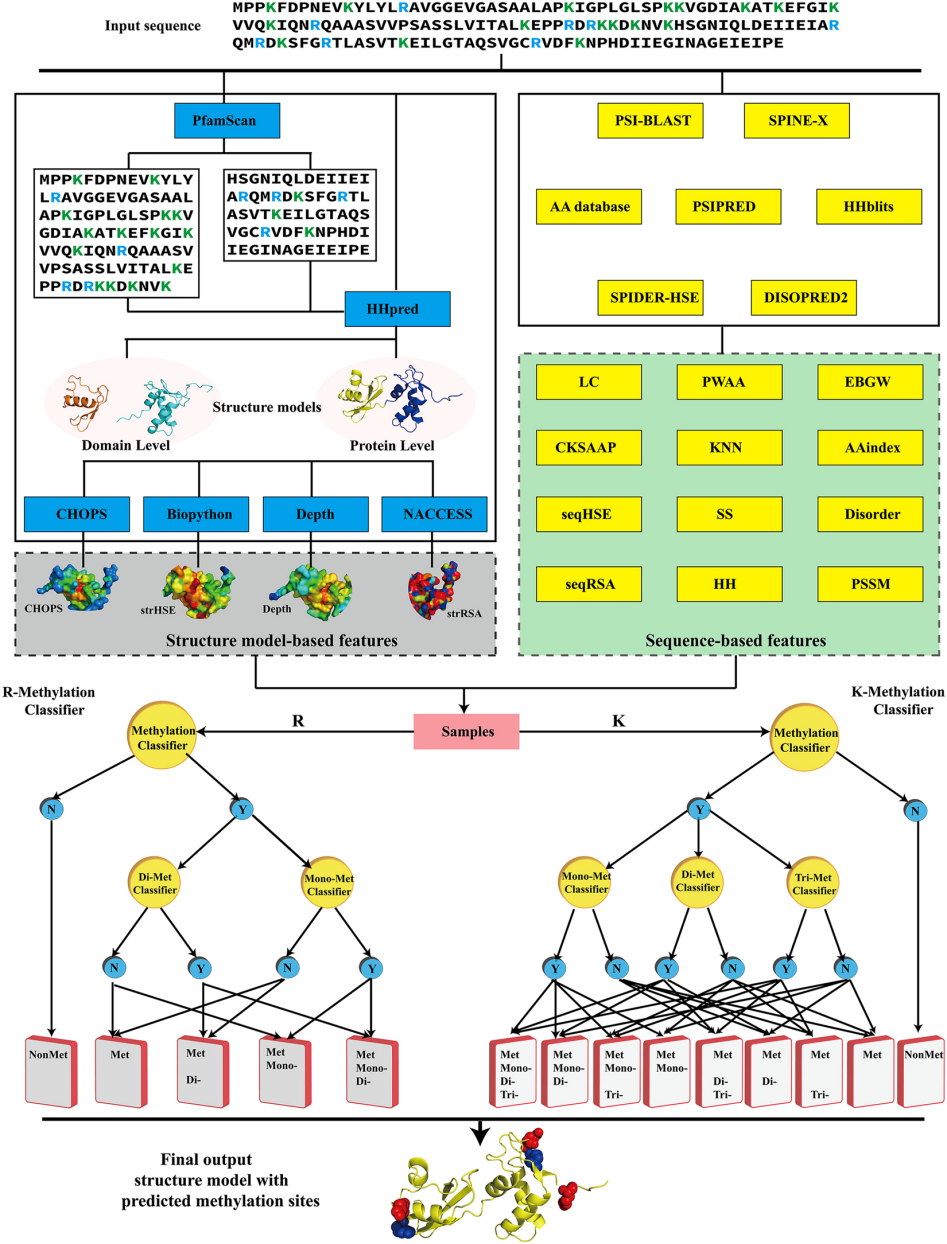
The flowchart of the methylation site and type prediction for arginine and lysine. Met-predictor combines two types of features: sequence-based features and structure model-based features. An SVM based network is built to simultaneously predict methylation sites as well as the methylation types of lysine and arginine.

Aiming to address these limitations, we proposed a novel tool, Met-predictor, to predict methyllysine, methylarginine, and their methylation types from protein sequence. We introduce two groups of features, sequence-based features and structure model-based features, for the methylation prediction. The sequence-based features include amino acid compositions and K-spaced amino acid pair compositions, which are calculated directly from protein sequences, as well as disorders, solvent accessible surface areas, secondary structures, and position-specific profiles, which are predicted from protein sequences using corresponding third-party tools^9,26–31^. The structure model-based features are geometric information extracted from predicted protein tertiary structure models, which are firstly used in the prediction of methylation sites. With these features, a support vector machine (SVM) based network is built to predict the lysine and arginine methylation sites and their methylation types (Fig. 2). First, we collected highly accurate experimental protein structure data for both lysine and arginine methylation sites to analyse the geometric information and structural properties around methylation sites, which demonstrated that the structure model-based features should be critical to the improvement of the methylation prediction. Furthermore, a large variety of sequence data was collected and divided into the training set for training models and the independent test sets for testing the predictive performance of the Met-predictor method. We compared several distinct classifiers and employed the feature selection procedure and the sliding window optimization strategy in order to improve the overall predictive performance. The Met-predictor approach was compared with the existing state-of-the-art methods on the independent test sets. The results indicated that the Met-predictor method is highly competitive for both arginine and lysine methylation predictions, especially when the structure model-based features were added. Met-predictor and all data are available at https://sourceforge.net/projects/met-predictor.

## Methods

### Sequence dataset

Given the difficulty of solving an experimental protein structure, we can see far more protein sequences in Uniprot^32^ than known structures in the Protein Data Bank^33^ (PDB). As this gap dramatically increased, we built a sequence dataset to test the performance of the Met-predictor, so that we can include enough updated and non-redundant methylation sites for model training.

All data is extracted from the UniProtKB/Swiss-Prot database (www.uniprot.org), PhosphoSitePlus (www.phosphosite.org) and dbPTM (http://dbptm.mbc.nctu.edu.tw/), for both lysine and arginine methylation sites. To remove the highly-homologous sequences and avoid such overestimation of the prediction accuracy of built models, we clustered the protein sequences with a threshold of 30% identity level using CD-HIT^34^. Finally, 20% of lysine or arginine data was randomly selected as an independent test set to compare performance with other existing predictors, while the other 80% was used as the training set for the training models. Recently, we found the dbPTM 2019 database^35^ was updated and accessible, where more experimentally validated PTMs from available databases and through manual curation of literature are integrated. Therefore, we further collected the methylation data from dbPTM 2019 database, and removed 30% sequence identity with our already-built training and test set. Additionally, we excluded the redundancy with the training sets of other state-of-the-art methods, such as, iLM_2L, MethK and GPS-MSP, which are used in this study for comparison. This new dataset includes a variety of newly determined methylation sites that have not been used in any existing methods for training and testing. We also notice that there are a large number of methylation sites and mono-methylation sites in the new dataset, but the numbers of di- and tri-methylation sites are insufficient. For example, the number of tri-methylation sites for lysine in the new dataset is only 6% of the training set. Thus, we use the data built from the dbPTM 2019 database as an independent test set II for an objective and fair comparison with other existing methods.

Table 1 shows the statistics of the final datasets for both lysine and arginine methylation. In total, we collected 5894 experimentally verified lysine methylation sites from 2849 protein sequences, and 5005 experimentally verified arginine methylation sites from 2723 protein sequences. Negative samples were randomly selected from all arginine and lysine residues that were not marked by any methylation information on the same proteins, with a ratio of 1:1 of positive versus negative sites, because the positive samples (methyllysine or methylarginine residues) in the datasets are considerably less than the negative samples. Different types of methylation (mono- and di- for arginine, and mono-, di-, and tri- for lysine) were also taken into account in data collection (Table 1) and further analysed. Detailed descriptions of sequence dataset construction are listed in the Text S1 of Supplement Information.

**Table 1:** Statistics of the lysine and arginine methylation datasets, covering different types of methylation (mono- and di- for arginine, and mono-, di-, and tri- for lysine).

Dataset	Methylation types	Methyllysine proteins/sites	Methylarginine proteins/sites
Training set	mono-	313/465	598/883
	di-	123/172	285/479
	tri-	88/117	—
	Total	485/721	818/1278
Independent test set I	mono-	77/110	159/231
	di-	30/45	69/103
	tri-	27/32	—
	Total	121/180	205/311
Independent test set II	mono-	2239/4973	206/323
	di-	7/21	110/217
	tri-	6/6	—
	Total	2243/4993	1700/3416
Structure dataset	Total	151/218(#3313)	99/128(#1515)

The training set is used for training models, while the two independent test sets are used for an objective and fair comparison with other existing methods. The experimental structure dataset is collected for analysing structure information of the methylation sites. The number with # indicates the number of negative samples.

### Structure dataset

Although there is insufficient structure data as listed in the PDB, we also collected highly-accurate experimental structure data from the PDB for both lysine and arginine methylation sites in order to analyse the usefulness of the structure model-based features of the methylation sites. Although the structure dataset is relatively small (Table 1), it is enough to make a significance test and demonstrate the usefulness of the structure model-based features. Note that this structure dataset is only used for feature analysis, rather than for model training or testing. The steps of structure dataset construction are as follows:

#### Step 1: Data collection

Based on the sequence dataset, we mapped the Uniprot IDs to PDB IDs from the Uniprot website, to get 5918 and 6152 structures from the PDB. For each Uniprot ID, all of its corresponding X-ray structures with resolution better than 3 Å in PDB were selected. Here, we got 4999 and 4900 structures for the lysine methylation dataset and the arginine methylation dataset, respectively. We then used blastclust^9^ to generate 485 clusters for the lysine methylation dataset and 511 clusters for the arginine methylation dataset with a sequence identity cut-off of 30%. In each cluster, we selected the longest sequence as the representative sequence. We further removed all structures with a sequence length <50, ending with 429 and 520 structures for the lysine methylation dataset and the arginine methylation dataset, respectively.

#### Step 2: Methylation site annotation

In this step, we first mapped methylation site annotations to structures. Any methylation site in the lysine methylation sequence dataset but mutated into non-lysine residue in the structure was not annotated, and the same with the arginine methylation dataset. The structures were removed if: (a) Structures did not have the side-chain atom “NZ” in lysine residue or “NH1” and “NH2” in arginine residue; (b) Structures did not have any methylation annotated site. After the first step, we got 151 and 99 protein structures for the lysine methylation dataset and the arginine methylation dataset, respectively. These proteins cover 218 experimental methyllysine sites and 128 experimental methylarginine sites. Similar to many function site datasets, positive samples in the methylation site dataset are considerably less than negative samples. In the lysine methylation dataset, the radio of positive samples to negative samples was 1:15, and 1:12 in the arginine methylation dataset (Table 1).

### Features

In this study, we incorporated two types of features: sequence-based features and structure model-based features.

A variety of different sequence-based features are included to build methylation prediction models. The sequence-based features can be further divided into 12 subtypes^36^: location coding (LC), position weight amino acid composition (PWAA), encoding based on grouped weight (EBGW), composition of k-spaced amino acid pairs (CKSAAP), K nearest neighbours score (KNN), physicochemical and biochemical property from AAindex database (AAindex), secondary structure (SS), relative solvent accessibility predicted from protein sequences (seqRSA), disorder type (Disorder), half sphere exposure predicted from protein sequences (seqHSE), position-specific scoring matrix (PSSM), and evolutionary information from HHblits (HH). The LC, PWAA, EBGW, CKSAAP and KNN features are calculated directly from protein sequences. In detail, LC features show the location information of a residue; PWAA features give the position information of a residue in a sliding window; EBGW features indicate the hydrophobicity and charged property of residues; CKSAAP features represent the composition of k-spaced amino acid pairs; KNN features calculate the percentage of positives in the K nearest neighbours of a residue based on the training dataset. On the other hand, the AAindex, SS, seqRSA, Disorder, seqHSE, PSSM, and HH features are predicted by some third-party tools from protein sequences. In detail, AAindex features are physicochemical and biochemical properties of an amino acid extracted from AAindex database^30^; SS features are the predicted secondary structure by PSIPRED^26^; seqRSA features include the relative solvent accessibility and backbone torsion angles predicted by SPINE-X^28^; Disorder features represent natively disordered residues recognized by DISOPRED2^27^; seqHSE features are the half sphere exposure predicted by SPIDER-HSE^31^; PSSM features represent position-specific scoring matrix generated by PSI-BLAST^9^; HH features give the evolutionary information generated by HHblits^29^. Detailed descriptions are shown in Text S2 of Supplement information.

Furthermore, structure model-based features are introduced into the methylation prediction for the first time. The structure model-based features are extracted from the predicted structure models, rather than the experimental protein structures. This is because the number of experimentally determined protein structures is lagging far behind the number of protein sequence since the experimental determination of protein structures is time and money consuming. Therefore, the first step is to build the structure models from protein sequences. In detail, the PfamScan^37^ package is employed to automatically separate a whole sequence into several domains. Then, HHpred^38^ software, which is a fast protein tertiary structure predictor, is used to predict the structure models of the above domain fragments as well as the whole protein sequence. Finally, the structure model-based features are calculated directly from the predicted structure models from HHpred.

The structure model-based features can be divided into four subtypes: convex hull of protein surface (CHOPS), half sphere exposure calculated from predicted structure models (strHSE), residue and L_1_ depth (Depth), and relative solvent accessibility calculated from predicted structure models (strRSA). Specifically, the CHOPS^39^ features represent the convex hull of protein surfaces, which is a measure to evaluate the locations of atoms in a protein. For example, atoms locating on the protein surface, where it is easier to contact with other proteins, will have a lower CHOPS value, while atoms locating in pockets of the protein surface, where it is more difficult to contact with other proteins, will have a higher CHOPS value. The strHSE features are the half sphere exposure calculated from the predicted structures by Biopython^40,41^ tool. Half sphere exposure^42^, which is a measure to evaluate the solvent exposure of a protein, is defined by the number of C_α_ atoms in two half-spheres around a residue’s C_α_ atom, where one of the half-spheres corresponds to the side-chain’s neighbourhood, while the other half-sphere is in the opposite direction. The Depth features include the residue depth^43^ and L_1_ depth^44^. The depth of an atom in a protein is defined as the distance between the atom and the nearest surface water molecule. The strRSA features represent the relative solvent accessibility calculated by NACCESS^45^ based on the predicted structure models. The relative solvent accessibility is defined as the ratio of the accessible surface area of a residue, observed in its three-dimensional structure, to that observed in an extended tri-peptide conformation. Detailed definitions can be found in the Text S3 of Supplement Information.

### Feature selection method based on different subtypes of features

The feature selection is performed based on different subtypes of features to remove redundant features and improve prediction performance, since feature redundancy may lead to the disadvantageous impact on prediction. The used features in this study include sequence-based features and structure model-based features. These features can be further divided into 16 subtypes: LC, PWAA, EBGW, CKSAAP, KNN, AAindex, SS, seqRSA, Disorder, seqHSE, PSSM, and HH belonging to sequence-based features, as well as CHOPS, strHSE, Depth, and strRSA contained in structure model-based features.

First, the Pearson Correlation Coefficient (PCC) between the feature vector and the true classification label vector on the training set was used to rank each subtype of features. Thus, 16 PCC-ranked lists corresponding to 16 subtypes of features were generated. Then, for each subtype of features, a stepwise feature selection was employed based on the support vector machine (SVM) classifier. At each round of stepwise feature selection, the accuracy of 5-fold cross-validation on the training set was calculated. The next feature from the PCC-ranked list was added if the accuracy increased. Finally, 16 optimal feature sets corresponding to 16 subtypes of features were obtained. The 16 subsets of selected features were further integrated to form the final features used in the Met-predictor to build models for methylation prediction. Through this feature selection method, we can reduce the feature dimensions and maintain the diversity of features at the same time. Furthermore, an SVM-based network was built to predict methylation types. The parameters of the SVM classifier were trained using the grid selection tool in LibSVM^46^ on the training set. The flowchart of Met-predictor is shown in Fig. 2.

### Performance evaluation

In this study, Matthews’s correlation coefficient (MCC), accuracy (ACC), sensitivity (SEN) (also called recall), specificity (SPE), precision (PRE), prevalence-corrected precision (CPRE)^47^, area under the receiver-operating characteristic (ROC) curve (AUC), and area under the precision-recall curve (PRAUC), were applied to evaluate the performance of Met-predictor on the training set for building prediction models by the SVM classifier and on the independent test sets for comparing with other existing methods. Among them, AUC and PRAUC were calculated by the area under the ROC curve and precision-recall curve, which are commonly used to evaluate a classifier or method. In addition, MCC, ACC, SEN, SPE, PRE and CPRE are calculated by the following formulas:

1 $$MCC=\frac{TP\times TN-FP\times FN}{\sqrt{(TP+FP)(TP+FN)(TN+FP)(TN+FN)}}$$

2 $$ACC=\frac{TP+TN}{TP+TN+FP+FN}$$

3 $$SEN=\frac{TP}{TP+FN}$$

4 $$SPE=\frac{TN}{TN+FP}$$

5 $$PRE=\frac{TP}{TP+FP}$$

6 $$CPRE=\frac{SEN}{SEN+r\times (1-SPE)}$$

where *TP*, *TN*, *FP*, and *FN* are the numbers of true positives, true negatives, false positives, and false negatives, respectively. *r* is the expected ratio of negative to positive numbers in the real-world data, which is listed in the Table S1.

## Results

### Analysis of compositional biases around methylation sites

**Figure 3:**
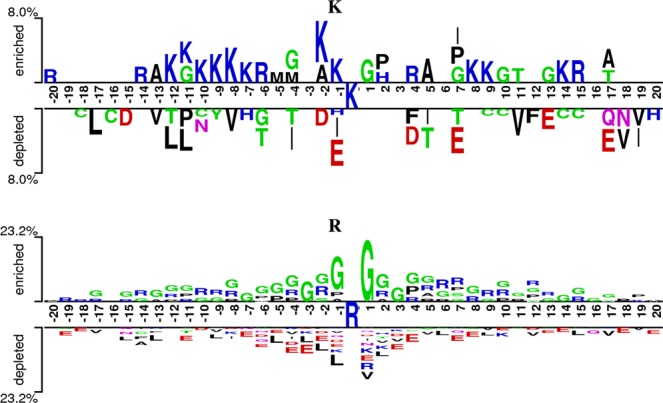
The compositional biases around the methylation sites compared to the non-methylation sites based on the two-sample logo. Only amino acid residues enriched or depleted around lysine methylation sites are shown. Each logo contains 41 residue fragments with 20 upstream and 20 downstream. Note that the scopes of the Y-axis for lysine and arginine are different. The lysine residue is represented by “K” while the arginine corresponds to “R”.

Two Sample Logo^48^ was adopted to show the compositional biases between methylation and non-methylation sites of lysine and arginine (Fig. 3). As shown in Fig. 3, there were substantial differences between lysine and arginine. The lysine (K) was enriched around lysine methylation sites, especially on the downstream, while the threonine (T), aspartic acid (D), and glutamic acid (E) were depleted around lysine methylation sites. For arginine methylation, the glycine (G) and arginine (R) were enriched at both upstream and downstream fragments, whereas leucine (L) and glutamic acid (E) were depleted around arginine methylation sites. Note that there is no side-chain for glycine (G). Therefore, the highly enriched glycine (G) around methylarginine may result in more opportunity for transferring a methyl group to the ε-Nitrogen atom of the side-chain of the arginine residue. Generally, the enriched residues and depleted residues had distinct differences between lysine and arginine methylation sites. Thus, building specific predictors for different methylated residues is necessary. More importantly, since there were considerable compositional biases between methylation sites and non-methylation sites for lysine and arginine, the sequence-based features, which incorporate the information from amino acid compositions, such as PWAA and CKSAAP features, should be useful for the methylation prediction.

### Analysis of structure model-based features on the experimental structure dataset

We have built an experimental structure dataset which contains 218 methyllysine sites and 128 methylarginine sites (Table 1) for analysing the effect of the structure model-based features, which include the geometric information and structural properties around methylation sites. Note that, here, the geometric information and structural properties were extracted directly from experimental protein structures, rather than extracted from predicted structure models, in order to obtain more accurate information for analyses.

Four types of structure model-based features, CHOPS, strHSE, Depth and strRSA, were calculated on the structure dataset.

**Figure 4:**
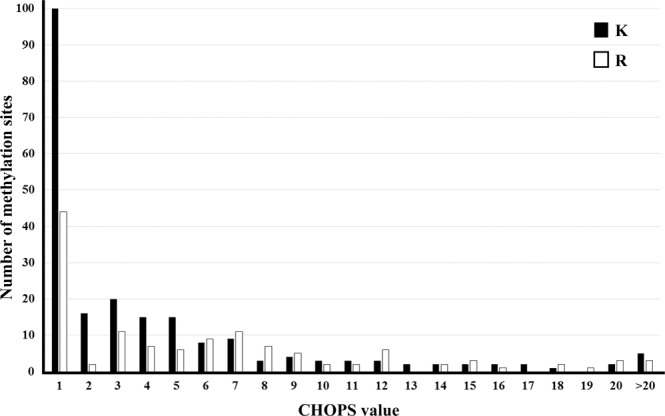
The CHOPS feature analysis for lysine and arginine methylation prediction. The bar represents the number of methylation samples on different CHOPS values for lysine and arginine methylation. For lysine, 46.1% (100/217) methylation samples have a CHOPS value of 1, which means these methylation samples are located at the first convex hull of the protein surface, and for arginine, there are 34.6% (44/127).

For the CHOPS features, Fig. 4 shows the distribution of methylation sites in each CHOPS value. CHOPS means the convex hull of protein surfaces. Atoms locating on the protein surface are easier to contact with atoms on other proteins, so that these atoms would have a lower CHOPS value. However, atoms locating in pockets of protein surface are more difficult to contact with atoms on other proteins, resulting in a higher CHOPS value. For lysine, 46.1% (100/217) of methylation sites had a CHOPS value of 1, which means these methylation sites were located at the first convex hull of the protein surface. For arginine, there were 34.6% (44/127) of methylation sites located at the first convex hull of the protein surface. For both lysine and arginine, the average CHOPS values for methylation samples were significantly lower than non-methylation samples (5.98 vs 7.62 for arginine and 4.40 vs 6.76 for lysine) with the *p*-values of 2E-03 and 4.57E-08. Tables S2 and S3 give the *p*-values calculated by the Student’s t-test or Wilcoxon signed-rank test, which is adopted based on whether the samples follow a normal distribution or not by Shapiro-Wilk test. The pairwise two-tailed test is made to test whether there are significant differences between positives and negatives. If there are significant differences, the pairwise one-tailed test is used to test whether the average value of one group is significantly higher or lower than the average value of the other group.

For the strHSE features, four kinds of HSE (HSEAU, HSEAD, HSEBU, and HSEBD, based on whether both the C_α_ and C_β_ positions (B) or only the C_α_ positions (A) are used, as well as whether the half-sphere is an up half-sphere (U) or a down half-sphere (D)) were calculated with different cut-off radius varying from 5Å to 30Å. As shown in Fig. S1, the HSEs of non-methyllysine were significantly greater than those of methyllysine with *p*-values all less than 8.0E-03 (*P*-values are shown in Tables S2 and S3), when the cut-off radius was greater than 10Å. For arginine, when the cut-off radius was greater than 25Å, there was a statistically significant difference between the HSEs of methylarginine and non-methylarginine with *p*-values all less than 2.4E-02 (*P*-values are shown in Tables S2 and S3).

For the Depth features, the L_1_ depths were calculated with the cut-off radius varying from 5Å to 30Å, and the results are shown in Fig. S2. These results indicated that both residue-level and atom-level L_1_ depths of methyllysine sites were significantly lower than those of non-methyllysine sites with *p*-values all less than 2.0E-02 (*P*-values are shown in Tables S2 and S3), when the cut-off radius was greater than 5Å. For arginine, a similar trend can be found when cut-off radius was greater than 20Å, which indicates that L_1_ depth is a good type of features for distinguishing methylation sites, especially for lysine.

For the strRSA features, the average RSA and side-chain RSA of methyllysine sites were 0.552 and 0.624, which were significantly greater than 0.513 and 0.578 for non-methyllysine sites with *p*-values of 5.0E-03 and 3.0E-03 (*P*-values are shown in Tables S2 and S3). For arginine, even though the RSAs of methylation sites were greater than those of non-methylation sites, there were no substantial differences.

Overall, these analyses on the experimental structure data indicated that structure model-based features can be useful to distinguish methylation sites from non-methylation sites. Therefore, we employed the structure model-based features into the support vector machine (SVM) classifier for training and independent testing in order to improve the prediction performance. However, in the training set and independent test sets, there are no experimental structures for most of proteins. Instead, we used HHpred^38^ to predict the structure model for each protein sequence, and calculated the structure model-based features based on the predicted structure models.

### The selection of classifier, feature, and sliding window size

First, we compared the performance of different classifiers on the 5-fold cross-validation of the training set. Here, the accuracy (ACC in Eq. (2)) was employed as the performance index to evaluate the performance of different classifiers. Tables S4 and S5 give the comparisons of performances based on the SVM, neural network, random forest and Bayes classifiers for lysine and arginine methylation prediction. For a fair comparison, the same features (all of the sequence-based and structure model-based features without performing the feature selection) were used for distinct classifiers to build models. The SVM classifier is implemented by LibSVM tool^46^, while the neural network, random forest and Bayes classifiers are implemented by Scikit-learn packages^49^ in Python with default settings. We found that the SVM classifier showed excellent performance for the prediction of methylation sites and methylation types. However, for lysine and arginine methylation sites prediction, the neural network and random forest classifiers performed poorly with accuracies less than 0.5 for most of sliding window sizes. The Bayes classifier was interior to the SVM classifier on the prediction of methylation types. Therefore, the SVM classifier was used in Met-predictor to build models for the independent tests with other existing methods. Note that the accuracy values of random forest and neural network classifiers on di- and tri-methylation prediction were relatively high. This is due to the fact that there are fewer samples for the di- and tri-methyllysine (172 for di- and 117 for tri-methyllysine as shown in Table 1) but much higher dimension of features (more than two thousand as shown in Table S6), which may lead to the over-fittings by random forest and neural network classifiers.

To avoid over-fitting in model training, we made the feature selection based on the feature selection method described in the “Feature selection method based on different subtypes of features” section. Tables S7 and S8 show the performance of the feature selection method with different sliding window sizes for lysine and arginine methylation prediction on the training set. When the feature selection was performed, the accuracy values were improved at least 8.7%, 4.7%, 8.1%, 8.6% and 1.1% for methylarginine, mono-methylarginine, di-methylarginine, methyllysine and mono-methyllysine prediction among different sliding window sizes. However, for di- and tri-methyllysine prediction, the feature selection reduced the accuracy performance because of the over-fitting of model training based on all of the features, where there were fewer samples for the di-and tri-methyllysine. Thus, the feature selection method is required to avoid over-fitting in model training and improve the performance of methylation prediction. Furthermore, the feature selection method can reduce the dimension of features, which would speed up the methylation prediction and improve efficiency. As shown in Table S6, the dimension of features reduced from 5013 to 154 for lysine and 155 for arginine.

**Figure 5:**
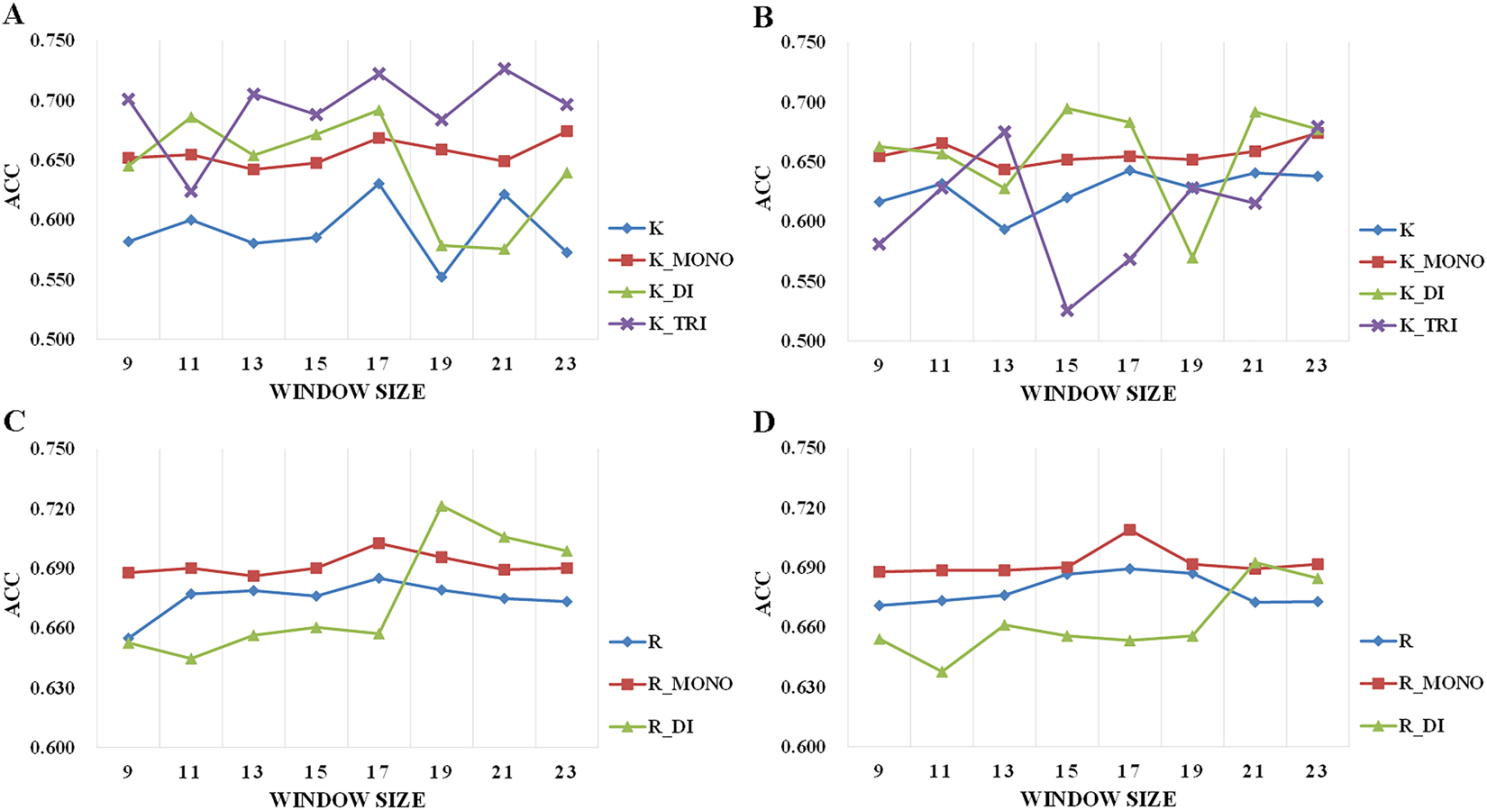
Predicted accuracy (ACC) of 5-fold cross-validation based on the training dataset for sliding window size ranging from 9 to 23. (**A**,**B**) Lysine methylation prediction based on sequence-based features and based on sequence-based features and structure model-based features. (**C**,**D**) Arginine methylation prediction sequence-based on sequence-based features and based on sequence-based features and structure model-based features.

Since different sliding windows may have distinct prediction performances, optimization of the sliding window sizes is required for selecting features and training models. The sliding windows were considered ranging from size 9 to 23, which covers all sliding window sizes used by previous methods^10–15,24,25^ about the prediction of methylation sites and methylation types. The accuracy was also employed as the performance index to evaluate the performance of different sliding window sizes. For each sliding window size, the 5-fold cross-validation was utilized based on the support vector machine (SVM) classifier to build the model and calculate the predicted accuracy. Figure 5 shows the predicted accuracy of each model based on different sliding window sizes. The sliding window sizes with the highest accuracy were chosen. For both lysine and arginine methylation, the sliding window size of 17 was selected. For mono-methylation prediction, the sliding window size of 17 was also selected for arginine, while 23 was selected for lysine. Overall, for both lysine and arginine, different sliding window sizes had stable accuracy values for methylation site and mono-methylation. But for di-and tri-methylations, the accuracies fluctuated strongly among different sliding window sizes.

### Prediction performance on training dataset

The predicted performances of the 5-fold cross-validation on the training set are shown in Table 2. For lysine and arginine methylation site prediction, the addition of structure model-based features reached a relatively better performance with the AUCs of 0.692 and 0.752 than utilization of only sequence-based features with the AUCs of 0.679 and 0.749. Furthermore, for the prediction of methylation types (mono-, di-, or tri-methylation), there were no considerable improvements for mono-methyllysine and di-methyllysine site prediction when adding the structure model-based features, whereas the AUC of tri-methyllysine site prediction largely improved from 0.611 to 0.754. For the prediction of arginine methylation types, the addition of structure model-based features improved the prediction performance of mono-methylarginine, but decreased the performance of di-methylarginine. These results indicated that Met-predictor incorporating both sequence-based and structure model-based features has a relatively better predictive ability on the training set.

**Table 2:** The prediction performance of the Met-predictor based on 5-fold cross-validation on the training set.

Residue	Methods	Window Size	MCC	ACC	SEN	SPE	PRE	CPRE	AUC	PRAUC
K	Met-predictor(seq)	17	0.261	0.630	0.655	0.606	0.624	0.061	0.679	0.669
	MONO	23	0.206	0.674	0.927	0.215	0.682	0.682	0.674	0.789
	DI	17	0.384	0.692	0.674	0.709	0.699	0.421	0.752	0.746
	TRI	21	0.455	0.726	0.769	0.684	0.709	0.320	0.779	0.746
	Met-predictor(seq + str)	17	0.286	0.643	0.662	0.624	0.638	0.064	0.692	0.685
	MONO	23	0.207	0.674	0.925	0.219	0.683	0.683	0.676	0.792
	DI	15	0.390	0.695	0.686	0.703	0.698	0.420	0.756	0.724
	TRI	23	0.359	0.679	0.692	0.667	0.675	0.287	0.754	0.749
R	Met-predictor(seq)	17	0.371	0.685	0.654	0.716	0.697	0.089	0.749	0.759
	MONO	17	0.148	0.703	0.992	0.056	0.701	0.701	0.606	0.761
	DI	19	0.377	0.721	0.409	0.909	0.729	0.729	0.745	0.665
	Met-predictor(seq + str)	17	0.380	0.689	0.642	0.737	0.709	0.094	0.752	0.763
	MONO	17	0.184	0.709	0.981	0.101	0.709	0.709	0.636	0.774
	DI	21	0.302	0.692	0.326	0.912	0.690	0.690	0.712	0.607

Two versions of Met-predictor are included here: Met-predictor(seq), where only sequence-based features are used to build models, and Met-predictor(seq + str), which uses not only sequence-based features, but also the novel structure model-based features to build models. The lysine residue is represented by “K” while the arginine corresponds to “R”. The definitions of measures MCC, ACC, SEN, SPE, PRE, CPRE, AUC and PRAUC are shown in “Performance Evaluation” section and Eqs. (1) to (6).

### Does the combination of sequence-based features with structure model-based features work?

Based on the above analyses on the structure dataset, we found the structure model-based features extracted directly from experimental protein structures can help distinguish the methylation sites from non-methylation sites. However, in the training set and the independent test sets, there are no experimental structures for most of proteins. Therefore, the structure model-based features were extracted from the structure models predicted by HHpred^38^. Here, we analysed the contribution of structure model-based features extracted from the predicted structure models to methylation prediction. Furthermore, we examined the effectiveness and rationality of combining sequence-based features with structure model-based features for methylation prediction. All analyses here are based on the independent test set I.

**Figure 6:**
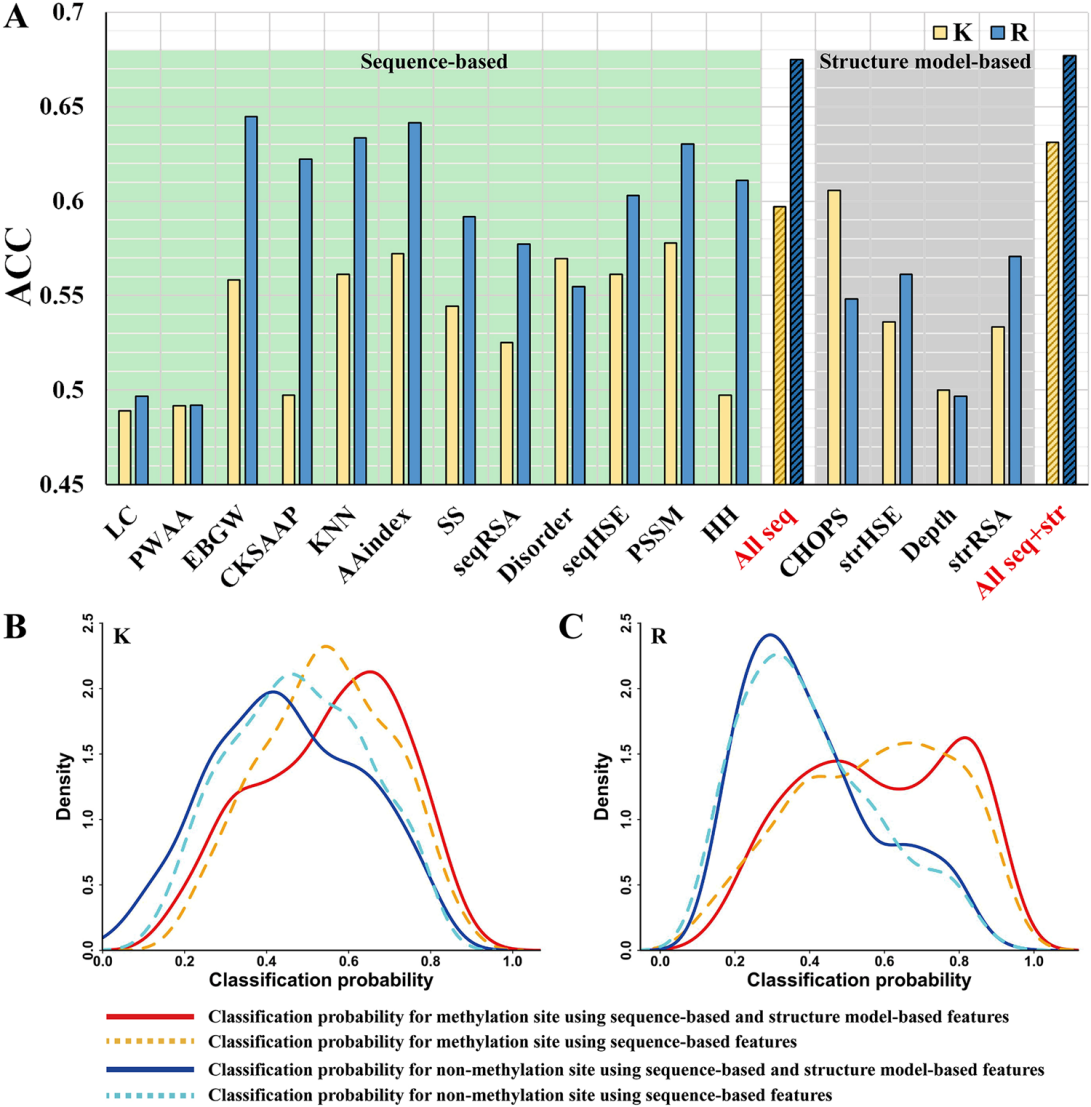
Predictive capability analysis of different types of features. (**A**) The prediction accuracy (ACC) of different models based on 16 subtypes of features, all sequence-based features, and all sequence-based and structure model-based features for lysine and arginine. The bar labelled “All seq” corresponds to the accuracy of model built by only sequence-based features, while the bar labelled “All seq + str” represents the accuracy of model built by both sequence-based and structure model-based features. For lysine, the CHOPS features have the highest accuracy, while for arginine the EBGW features are the best. (**B**) The distributions of classification probabilities for methyllysine and non-methyllysine sites. (**C**) The distributions of classification probabilities for methylarginine and non-methylarginine sites. The lysine residue is represented by “K” while the arginine corresponds to “R”.

Figure 6A and Table S9 show the predictive capabilities of 16 subtypes of features (LC, PWAA, EBGW, CKSAAP, KNN, AAindex, SS, RSA, Disorder, HSE, PSSM, HH, CHOPS, strHSE, Depth, and strRSA, which are described in the “Features” section). 16 models were trained on the training set based only on the corresponding subtype of features using an SVM classifier, respectively. Then, the accuracy values (ACC in Eq. (2)) on the independent test set I were calculated to evaluate the performance of each subtype of features.

For sequence-based features, the model based on KNN features or EBGW features had the accuracy values of 0.561 and 0.558 for lysine as well as 0.633 and 0.645 for arginine, which outperformed other sequence-based features, while the accuracies of the LC and PWAA features were relatively poor (0.489 and 0.492 for lysine, 0.497 and 0.492 for arginine). Interestingly, CKSAAP features had relatively higher accuracy value of 0.622 for arginine but performed poorly for lysine (0.497). As shown in Fig. 3, there were substantial compositional biases between methylation sites and non-methylation sites of lysine and arginine (for example, the lysine (K) was enriched especially on the downstream for lysine methylation; the glycine (G) and arginine (R) were enriched at both upstream and downstream for arginine methylation). Thus, the residue composition-related features, KNN, EBGW, and CKSAAP, can reach relatively good performances. On the other hand, the AAindex features had relatively higher accuracy values of 0.572 and 0.642 for both lysine and arginine methylation site prediction. In contrast, the Disorder features only performed well for lysine with an accuracy of 0.569. We further combined all sequence-based features to build the model and evaluate the prediction performance. We found the integration of all sequence-based features can considerably improve the predictive performance for both lysine and arginine methylation, reaching the accuracy values of 0.597 and 0.675.

For structure model-based features, CHOPS features had the highest accuracy of 0.606 among all 16 subtypes of features, which are even higher than the accuracy of all sequence-based features of 0.597, for lysine methylation prediction. Thus, we combined all sequence-based features with all structure model-based features to build a final model. The prediction accuracy values of 0.631 and 0.677 based on models integrating both sequence-based and structure model-based features were superior to the accuracy values of 0.597 and 0.675 based only on sequence-based features, for both lysine and arginine methylation, which indicates that the addition of structure model-based features can substantially improve the prediction performance, especially for lysine methylation.

We present in Fig. 6B,C the distributions of classification probabilities for methylation and non-methylation sites to further investigate why the addition of structure model-based features can improve the performance. The classification probability is the predictive probability of classifying a sample as the methylation site. Thus, a good prediction method should have larger classification probabilities for positive samples, while lower classification probabilities for the negative sample. For lysine methylation prediction, the peak of the density curve of methylation probabilities moved to the right (i.e., the average probability improved from 0.545 to 0.553), while the peak of non-methylation probabilities moved to the left (i.e., the average probability improved from 0.480 to 0.451), when the structure model-based features were added, indicating the usefulness of structure model-based features. Furthermore, we found the addition of the structure model-based feature reduced overlapped area between the density curves of methylation and non-methylation probabilities (i.e., the overlapped area between two solid lines was smaller than the overlapped area between two dashed lines in Fig. 6B), indicating that it may be easier to distinguish methyllysine sites from non-methyllysine sites with the help of structure model-based features. Similarly, for arginine, the addition of the structure model-based features made the peak of the density curve of methylation probabilities move to the right (i.e., the average probability improved from 0.568 to 0.585). Table S10 shows the statistical significance test on the ability of methylation prediction between the two groups of features. The combination of sequence-based and structure model-based features had significantly lower average probability of non-methyllysine of 0.451, as well as significantly larger average probability of methylarginine of 0.585, than the use of only sequence-based features (0.480 and 0.568), with *p*-values of 5.0E-04 and 1.1E-05 calculated by pairwise one-tailed Wilcoxon signed-rank tests, indicating the combination of sequence-based features and structure model-based features can be more helpful for both lysine and arginine methylation predictions.

### Performance on independent test sets and comparison with existing methods

Aiming to further evaluate the performance of the Met-predictor, we compared the Met-predictor with other existing methods, including MEMO^10^, MASA^11^, PLMLA^12^, PmeS^13^, MethK^14^, iLM_2L^15^ and GPS-MSP^25^ on two independent test sets. These tools or servers were run with their default settings. The comparison results on the two independent test sets are shown in Tables 3 and 4. Matthews’s correlation coefficient (MCC), accuracy (ACC), sensitivity (SEN), specificity (SPE), precision (PRE), prevalence-corrected precision (CPRE), area under the receiver-operating characteristic curve (AUC), and area under the precision-recall curve (PRAUC) were applied to evaluate the performance of methylation prediction (Eqs. (1) to (6)). Note that the probabilities of both positive and negative samples are needed to calculate the AUC and PRAUC values. Therefore, we cannot give the AUC and PRAUC values of other methods for comparison, because other tools or servers do not output the probabilities for their predicted sites or only gives the probabilities of positive sites they predicted.

**Table 3:** Performance comparison of the Met-predictor with other existing methods on the independent test set I.

Residue	Methods	MCC	ACC	SEN	SPE	PRE	CPRE	AUC	PRAUC
K	MEMO	0.104	0.528	0.106	0.950	0.679	0.075	—	—
	MASA	0.164	0.531	0.067	0.994	0.923	0.301	—	—
	PLMLA	0.061	0.531	0.517	0.544	0.531	0.042	—	—
	PmeS	0.209	0.542	0.083	1.000	1.000	1.000	—	—
	MethK	0.101	0.558	0.019	1.000	1.000	1.000	—	—
	iLM_2L	0.075	0.531	0.239	0.822	0.573	0.049	—	—
	MONO	0.039	0.450	0.218	0.814	0.649	0.648	—	—
	DI	0.151	0.522	0.044	1.000	1.000	1.000	—	—
	TRI	0.291	0.578	0.156	1.000	1.000	1.000	—	—
	GPS-MSP	0.130	0.517	0.033	1.000	1.000	1.000	—	—
	MONO	−0.094	0.383	0.000	0.986	0.000	0.000	—	—
	DI	0.106	0.511	0.022	1.000	1.000	1.000	—	—
	TRI	Nan	0.500	0.000	1.000	Nan	Nan	—	—
	Met-predictor(seq)	0.195	0.597	0.633	0.561	0.591	0.053	0.611	0.606
	MONO	0.126	0.617	0.845	0.257	0.641	0.641	0.594	0.705
	DI	0.223	0.611	0.578	0.644	0.619	0.351	0.557	0.553
	TRI	0.194	0.594	0.469	0.719	0.625	0.265	0.611	0.549
	Met-predictor(seq + str)	0.261	0.631	0.644	0.617	0.627	0.061	0.655	0.647
	MONO	0.136	0.622	0.864	0.243	0.642	0.642	0.587	0.699
	DI	0.291	0.644	0.578	0.711	0.667	0.400	0.660	0.601
	TRI	0.221	0.609	0.531	0.688	0.630	0.269	0.664	0.585
R	MEMO	0.282	0.624	0.386	0.862	0.736	0.104	—	—
	MASA	0.316	0.622	0.305	0.939	0.833	0.172	—	—
	PmeS	0.176	0.587	0.498	0.675	0.605	0.060	—	—
	GPS-MSP	0.192	0.550	0.122	0.977	0.844	0.181	—	—
	MONO	0.113	0.293	0.048	1.000	1.000	1.000	—	—
	DI	0.024	0.505	0.049	0.961	0.556	0.384	—	—
	Met-predictor(seq)	0.352	0.675	0.637	0.714	0.690	0.085	0.723	0.731
	MONO	0.097	0.746	1.000	0.013	0.745	0.745	0.541	0.755
	DI	0.073	0.633	0.223	0.837	0.404	0.404	0.583	0.579
	Met-predictor(seq + str)	0.355	0.677	0.630	0.723	0.695	0.086	0.734	0.746
	MONO	0.126	0.746	0.978	0.075	0.753	0.753	0.574	0.778
	DI	0.122	0.662	0.194	0.894	0.476	0.475	0.628	0.617

Two versions of Met-predictor are included here: Met-predictor(seq), where only sequence-based features are used to build models, and Met-predictor(seq + str), which uses not only sequence-based features, but also the novel structure model-based features to build models. The lysine residue is represented by “K” while the arginine corresponds to “R”. The definitions of measures MCC, ACC, SEN, SPE, PRE, CPRE, AUC and PRAUC are shown in “Performance Evaluation” section and Eqs. (1) to (6). The “Nan” for the MCC, PRE and CPRE is because both TP and FP are zero, resulting in the measure divided by zero. There are no AUC and PRAUC values for other methods because other tools or servers do not output the probabilities for their predicted sites or only gives the probabilities of positive sites they predicted. Note that the probabilities of both positive and negative samples are needed to calculate the AUC and PRAUC values.

**Table 4:** Performance comparison of the Met-predictor with other existing methods on the independent test set II.

Residue	Methods	MCC	ACC	SEN	SPE	PRE	CPRE	AUC	PRAUC
K	MASA	0.014	0.501	0.010	0.992	0.574	0.057	—	—
	PLMLA	0.067	0.533	0.540	0.527	0.533	0.048	—	—
	PmeS	−0.018	0.499	0.004	0.993	0.382	0.027	—	—
	MethK	0.007	0.500	0.001	1.000	0.667	0.082	—	—
	iLM_2L	−0.005	0.498	0.213	0.783	0.495	0.042	—	—
	MONO	−0.018	0.493	0.200	0.786	0.483	0.996	—	—
	DI	Nan	0.500	0.000	1.000	Nan	Nan	—	—
	TRI	0.258	0.625	0.500	0.750	0.667	0.002	—	—
	GPS-MSP	−0.010	0.499	0.002	0.997	0.400	0.029	—	—
	MONO	0.004	0.500	0.001	0.999	0.571	0.997	—	—
	DI	Nan	0.500	0.000	1.000	Nan	Nan	—	—
	TRI	Nan	0.500	0.000	1.000	Nan	Nan	—	—
	Met-predictor(seq)	0.035	0.517	0.508	0.526	0.518	0.046	0.520	0.513
	MONO	0.006	0.503	0.488	0.518	0.503	0.996	0.506	0.500
	DI	0.000	0.500	0.364	0.636	0.500	0.004	0.504	0.512
	TRI	0.258	0.625	0.500	0.750	0.667	0.002	0.625	0.748
	Met-predictor(seq + str)	0.109	0.554	0.532	0.576	0.557	0.053	0.566	0.540
	MONO	0.094	0.545	0.412	0.678	0.562	0.997	0.560	0.538
	DI	0.218	0.545	0.091	1.000	1.000	1.000	0.570	0.547
	TRI	0.577	0.750	0.500	1.000	1.000	1.000	0.625	0.748
R	MASA	Nan	0.500	0.000	1.000	Nan	Nan	—	—
	PmeS	0.070	0.516	0.074	0.958	0.640	0.064	—	—
	GPS-MSP	0.113	0.531	0.115	0.948	0.688	0.078	—	—
	MONO	0.232	0.586	0.249	0.923	0.764	0.252	—	—
	DI	0.091	0.534	0.207	0.862	0.600	0.092	—	—
	Met-predictor(seq)	0.166	0.583	0.634	0.532	0.575	0.050	0.640	0.666
	MONO	0.370	0.680	0.793	0.568	0.647	0.161	0.749	0.736
	DI	0.044	0.506	0.023	0.989	0.667	0.119	0.530	0.528
	Met^−^predictor(seq + str)	0.262	0.618	0.404	0.833	0.707	0.085	0.643	0.677
	MONO	0.412	0.645	0.290	1.000	1.000	1.000	0.705	0.727
	DI	0.132	0.517	0.034	1.000	1.000	1.000	0.632	0.606

Table layout and description are identical to Table 3.

On the independent test set I (Table 3), Met-predictor incorporating both sequence-based and structure model-based features had 13.1% and 8.5% higher ACC than the best of other methods for methyllysine and methylarginine prediction. Specifically, for lysine methylation site prediction, the Met-predictor based on only the sequence-based features had slightly lower MCC than PmeS method (0.195 vs 0.209). However, the addition of structure model-based features had considerably improved the predictive performance of the model from MCC of 0.195, ACC of 0.597 and AUC of 0.611 to 0.261, 0.631 and 0.655, respectively, which were considerably higher than all other methods. For arginine methylation site prediction, the Met-predictor method based only on the sequence-based features had already performed considerably better than other methods on MCC, ACC and SEN, but the addition of structure-based features still improved the predictive performance of arginine methylation. However, the SPE, PRE and CPRE of Met-predictor on both lysine and arginine methylation prediction were worse than PmeS, MethK and GPS-MSP. This is because PmeS, MethK and GPS-MSP methods tended to predict a tiny number of methylation sites and the small portion of predictions was correct, i.e., the true positives (TP) were very small while false positives (FP) were zero. In the existing methods, iLM_2L and GPS-MSP had the ability to build models for determining the methylation types. When compared with iLM_2L, which was designed for only lysine methylation prediction, the ACC and SEN of the Met-predictor with both sequence-based and structure model-based features were higher on all three types, and the MCC was higher on both mono- and di-methyllysines. Similarly, iLM_2L method had higher SPE, PRE and CPRE values for di- and tri-methylation prediction, because these two datasets were very small (including only 45 true di-methyllysines and 32 true tri-methyllysines), so that this method predicted a very small but accurate number of di- or di-methyllysines (i.e., the true positives (TP) were very small while false positives (FP) were zero). GPS-MSP method, which can make prediction of methylation types for both lysine and arginine, performed poorly on mono- and tri-methyllysine prediction, which is due to the fact that the true positives (TP) and false positives (FP) predicted by GPS-MSP were extremely small and even zero (“Nan” values indicated that both TP and FP were zero).

The independent test set II was collected from a recently released database with a variety of newly determined methylation sites which have not been used in any existing methods for training and testing. Furthermore, the redundancy with the training sets of other state-of-the-art methods was excluded. Therefore, the comparison with other existing methods on this dataset should be more objective and fairer. Table 4 shows the performance comparison of the Met-predictor with other existing methods on the independent test set II. Note that the MEMO method was not included on the independent test set II, because it is inaccessible after we collected the independent test set II. Similar results with the independent test set I can be found. But the performance of each method on the independent test set II reduced when compared with independent test set I, which is because the independent test set II was brand new so that the over-fitting of model training was avoided. The performance of Met-predictor incorporating both sequence-based and structure model-based features on tri-methyllysine prediction was considerably excellent, because there was an extremely small number of tri-methyllysine samples (only 6 as shown in Table 1). Overall, the Met-predictor outperformed other methods on MCC, ACC and SEN for methylation sites prediction and their methylation types.

## Discussion

In this study, a novel tool, Met-predictor, is developed for largely improving the performance to identify lysine and arginine methylation sites and their methylation types. Met-predictor combines a novel group of structure model-based features with an SVM based network to simultaneously predict methylation sites, as well as the methylation types (i.e., mono-, di- and tri-methylation) of lysine and arginine. Moreover, a classifier evaluation, an effective feature selection method, and a sliding window optimization strategy are employed in order to improve prediction performance.

In the predictive capability analyses of different subtypes of features, the largest contribution came from structure model-based features for lysine methylation prediction and sequence-based features for arginine methylation prediction. For lysine especially, all four kinds of geometric information from predicted protein structures (i.e., structure model-based features CHOPS, strHSE, Depth, strRSA) had relatively good performances to distinguish positive samples from negative samples. This may be due to the fact that the nitrogen atom in a lysine residue’s side-chain will have more opportunity to contact to a methyl group donor when the lysine residue has less surrounding atoms (the values of strHSE are small) and is located at a convex region (the values of CHOPS are small). When combining the sequence-based features with the structure model-based features, the Met-predictor showed improved prediction performance on two independent test sets.

Furthermore, the proposed method considerably improved the prediction of both lysine and arginine methylation sites when compared with existing methods. In comparison to iLM_2L and GPS-MSP, which were the only existing methods for identifying different methylation types, the higher performance was attained by the Met-predictor for mono- and di-methylation site prediction. In the future, we will consider the employment of some new types of features and more effective feature selection methods, which may hopefully assist in making progress in the prediction of arginine methylation sites and types.

## Supplementary information

Supplementary information.
