# Supplementary material for: Two-Level Protein Methylation Prediction using structure model-based features
Source: Sci Rep. 2020 Apr 7;10:6008. doi: 10.1038/s41598-020-62883-2 (PMC7138832; doi:10.1038/s41598-020-62883-2)
Supplement: Supplementary file 1 — Supplementary information. [file 41598_2020_62883_MOESM1_ESM.docx]

**Two-Level Protein Methylation Prediction using**

**structure model-based features**

Wei Zheng^1,3†^, Qiqige Wuyun^2,3†^, Micah Cheng^4^, Gang Hu^3,^* and Yanping Zhang^5,^*

*^1^Department of Computational Medicine and Bioinformatics, University of Michigan, Ann Arbor, MI 48109 USA*

*^2^Computer Science and Engineering Department, Michigan State University, East Lansing, MI 48823 USA*

*^3^School of Mathematical Sciences and LPMC, Nankai University, Tianjin 300071, PR China*

*^4^Department of Electrical Engineering and Computer Science,* *University of Michigan, Ann Arbor, MI 48109 USA*

*^5^Department of Mathematics, School of Mathematics and Physics, Hebei University of Engineering, Handan 056038, PR China*

†These two authors contributed equally.

*Correspondence should be addressed to

Yanping Zhang,

Address: Hebei University of Engineering, Handan 056038, PR China

Phone: 15613093054

Fax: No

Email: zhangyanping@hebeu.edu.cn

Gang Hu,

Address: Nankai University, Tianjin 300071, PR China

Phone: 13116138510

Fax: No

Email: huggs@nankai.edu.cn

**Supplement Information**

**Supporting Texts**

- **Text S1.** Sequence dataset
- **Text S2.** Sequence-based features
- **Text S3.** Structure model-based features

**Supporting Tables**

- **Table S1.** Statistics of the number of positive and negative samples for lysine and arginine methylation datasets.
- **Table S2.** Statistical test between positive and negative samples on the structure dataset for arginine samples.
- **Table S3.** Statistical test between positive and negative samples on the structure dataset for lysine samples.
- **Table S4.** The comparison of the support vector machine (SVM), neural network (NN), random forest (RF) and Bayes classifiers with different sliding window sizes based on both sequence-based and structure model-based features for lysine (K) methylation prediction on the training set.
- **Table S5.** The comparison of the support vector machine (SVM), neural network (NN), random forest (RF) and Bayes classifiers with different sliding window sizes based on both sequence-based and structure model-based features for arginine (R) methylation prediction on the training set.
- **Table S6.** The dimensions of feature selection for the Met-predictor based on both sequence-based features and structure model-based features.
- **Table S7.** The performance of the feature selection method with different sliding window sizes based on both sequence-based and structure model-based features for lysine (K) methylation prediction on the training set.
- **Table S8.** The performance of the feature selection method with different sliding window sizes based on both sequence-based and structure model-based features for arginine (R) methylation prediction on the training set.
- **Table S9.** The prediction accuracy (ACC) of different models based on 16 subtypes of features, all sequence-based features, and all sequence-based and structure model-based features for lysine (K) and arginine (R).
- **Table S10.** Statistical test between the prediction probabilities based on two groups of features for positive and negative samples.

**Supporting Figures**

- **Figure S1.** Four types of HSE values for methyllysine/methylarginine and non-methyllysine/non-methylarginine sites with radius from 5Å to 30Å.
- **Figure S2.** The residue-level L_1_ depth and atom-level L_1_ depth for methyllysine/methylarginine and non-methyllysine/non-methylarginine sites with radius from 5Å to 30Å.

**References**

# **Supporting Texts**

**Text S1. Sequence Dataset**

Given the difficulty of solving an experimental protein structure, we can see far more protein sequences in Uniprot[^1^](#_ENREF_1), than known structures in the Protein Data Bank (PDB)[^2^](#_ENREF_2). As this gap dramatically increases, we built a sequence dataset to test the performance of our method, so that we can include enough updated and non-redundant methylation sites for model training.

All data are extracted from the UniProtKB/Swiss-Prot database (www.uniprot.org), PhosphoSitePlus (www.phosphosite.org) and dbPTM (http://dbptm.mbc.nctu.edu.tw/), for both lysine and arginine methylation sites. First, we obtained candidate proteins including experimental methylarginine sites by searching information containing ‘Omega-N-methylarginine’, ‘Omega-N-methylated arginine’, ‘N5-methylarginine’, ‘5-methylarginine’, ‘Dimethylated arginine’, ‘N2, N2-dimethylarginine’, ‘symmetric dimethylarginine’ and ‘asymmetric dimethylarginine’, and including experimental methyllysine sites through the keywords ‘N6, N6, N6-trimethyllysine’, ‘N6, N6-dimethyllysine’, ‘N6-methylated lysine’, ‘N6-methyllysine’ and ‘N6-poly(methylaminopropyl)lysine’. Then, experimentally verified methylated arginine and lysine sites were extracted as positive samples by excluding the annotation of ‘By similarity’, ‘Potential’, ‘Possible’ or ‘Probable’ in the description field. At last, a total of 153 proteins covering 459 experimental methylarginine sites and 247 proteins covering 702 experimental methyllysine sites were collected from the UniProtKB/Swiss-Prot database, respectively. Additionally, PhosphoSitePlus provides comprehensive information for protein post-translational modifications (PTMs), including phosphorylation, acetylation, methylation, ubiquitination, and so on. dbPTM integrates a total of eleven biological databases related to PTMs. We obtained 3136/247 proteins covering 7122/1192 experimental methylarginine sites and 1404/306 proteins covering 2378/1013 experimental methyllysine sites from the PhosphoSitePlus/dbPTM database. Then, we mapped all protein data to the UniProt database by retrieving the corresponding Uniprot IDs and integrated the extracted data from the above three databases by removing all identical sequences. To remove the highly-homologous sequences and avoid such overestimation of the prediction accuracy of built models, we clustered the protein sequences with a threshold of 30% identity level using CD-HIT[^3^](#_ENREF_3)^9^. Finally, 20% of lysine or arginine data was randomly selected as an independent test set to avert over-fitting for the training models and compare performance with other existing predictors, while the left 80% was used as the training set for the training models.

Recently, we found the dbPTM 2019 database[^4^](#_ENREF_4) was updated and accessible, where more experimentally validated PTMs from available databases and through manual curation of literature are integrated. Therefore, we further collected the methylation data from dbPTM 2019 database, and removed 30% sequence identity with our already-built training and test set. Additionally, we excluded the redundancy with the training sets of other state-of-the-art methods, such as, iLM_2L[^5^](#_ENREF_5), methK[^6^](#_ENREF_6) and GPS-MSP[^7^](#_ENREF_7), which are used in our study for comparison. This new dataset includes a variety of newly determined methylation sites which have not been used in any existing methods for training and testing. Thus, we use the data built from dbPTM 2019 database as an independent test II for an objective and fair comparison with other existing methods.

Negative samples were randomly selected from all arginine and lysine residues that were not marked by any methylation information on the same proteins, with a ratio of 1:1 of positive versus negative sites, because the positive samples (methyllysine or methylarginine residues) in the datasets are considerably less than the negative samples. Different types of methylation (mono- and di- for arginine, and mono-, di-, and tri- for lysine) were also taken into account in data collection and further analyzed.

**Text S2. Sequence-based features**

**Location Coding (LC)**

For the sites located in the N-terminal, C-terminal or the middle of a sequence, we use a 3-bit binary to encode this terminal information, i.e. N-terminal for 100, C-terminal for 001, middle for 010.

**Position Weight Amino Acid Composition (****PWAA)**

Position weight amino acid composition (PWAA)[^8^](#_ENREF_8)^,^[^9^](#_ENREF_9) is designed to avoid losing the sequence-order information of amino acid residues around certain sites. The position information of an amino acid in the sliding window can be calculated by the following formula:

$$C_{i}=\frac{1}{L(L+1)}\sum_{j=-L}^{L} x_{i,j}(j+\frac{\left|j\right|}{L})$$

where *L* denotes the number of upstream residues or downstream residues from the central site in the sliding window, *x_i,j_* = 1 if *a_i_* is the *j*-th residue in the sliding window, otherwise *x_i,j_* = 0.

**Encoding Based on Grouped Weight (****EBGW)**

Encoding based on grouped weight (EBGW)[^8-10^](#_ENREF_8) is an encoding scheme of the amino acid sequence based on the hydrophobicity and charged character of amino acid residues.

First, 20 amino acid residues are divided into four different classes as follows: hydrophobic group *C1*={*A,F,G,I,L,M,P,V,W*}, polar group *C2*= {*C,N,Q,S,T,Y*}, positively charged group *C3*={*H,K,R*}, and negatively charged group *C4* = {*D,E*}. Then, we calculate three binary sequences of a certain sliding window:

$$H1(a_{j})=\left\{\begin{matrix} 1\text{ }if\text{ }a_{j}\in C1\cup C2 \\ 0\text{ }if\text{ }a_{j}\in C3\cup C4 \end{matrix}\right.$$

$$H2(a_{j})=\left\{\begin{matrix} 1\text{ }if\text{ }a_{j}\in C1\cup C3 \\ 0\text{ }if\text{ }a_{j}\in C2\cup C4 \end{matrix}\right.$$

$$H3(a_{j})=\left\{\begin{matrix} 1\text{ }if\text{ }a_{j}\in C1\cup C4 \\ 0\text{ }if\text{ }a_{j}\in C2\cup C3 \end{matrix}\right.$$

where *a_j_* represents the *j*-th residue in the sliding window sequence.

For each binary sequence, we can calculate *K* feature values based on *K* sub-sequences increasing in length as follows:

$$X(k)=\frac{sum(k)}{Int(N\cdot k/K)},k=1,2,...,K$$

where the function *sum* (*k*) gives the number of 1 in the *k*-th sub-sequence, *Int*(*N*·*k*/*K*) denotes the length of the *k*-th sub-sequence, the *Int*() rounds a number to the nearest integer and *N* is the length of the sliding window sequence. Here, we make *K* = 5.

**Composition of k-spaced amino acid pairs (CKSAAP)**

The CKSAAP[^11^](#_ENREF_11) encoding scheme means the composition of *k*-spaced residue pairs in the sliding window. In this study, we take *k*=0. Therefore, there are 400 compositions of 0-spaced residue pairs, which can be calculated by

$$\left(\frac{N_{AA}}{N_{Total}},\frac{N_{AC}}{N_{Total}},\frac{N_{AC}}{N_{Total}},...,\frac{N_{YY}}{N_{Total}}\right)_{400}$$

where the *N_Total_* represents the total number of residue pairs in the sliding window, and the *N_XX_* is the number of the residue pair XX in the sliding window.

**K Nearest Neighbours (KNN) Score**

To take advantage of the cluster information of local sequence fragments for predicting methylation sites, we take a *K* nearest neighbours (KNN) score algorithm[^12^](#_ENREF_12)^,^[^13^](#_ENREF_13).

Firstly, we find the *K* nearest neighbours of a residue in both positive and negative datasets (training dataset was used in this paper). In detail, the distance between two local sequence fragments *S*_1_ and *S*_2_ defined as:

$$D(S_{1},S_{2})=1-\frac{\sum_{i=-n}^{n} sim(S_{1}(i),S_{2}(i))}{2L+1}$$

$$sim(a,b)=\frac{M(a,b)-\min(M)}{\max(M)-\min(M)}$$

where *a* and *b* are the two amino acid residues, *M* represents the substitution matrix of BLOSUM62^[14](#_ENREF_14)^, and the sliding window size is 2*L*+1.

After that, the corresponding KNN score is then extracted as follows: (i) Calculate the average distance from the sequence fragment *S* to the training dataset (contain the positive and negative datasets); (ii) Sort the neighbours by the distances and choose the *K* nearest neighbours; (iii) Calculate the percentage of positive neighbours in its *K* nearest neighbours as the KNN score.

Last, we choose five different *K* values, i.e., 1/2, 1/4, 1/8, 1/16 and 1/32 of the size of the training dataset.

**AAindex**

AAindex database^[15](#_ENREF_15)^ provides numerical indices that describe various physicochemical and biochemical properties of amino acids. For each index, we can express the physicochemical and biochemical information in the sliding window with 2*L*+1amino acid residues by the following formula:

$$A=\frac{1}{2L+1}\sum_{j=-L}^{L} p_{j}$$

where *p_j_* is the index value of the *j*-th residue in the sliding window.

Additionally, Atchley[^16^](#_ENREF_16) summarized five highly representative indices, i.e., electrostatic charge, codon diversity, molecular volume, secondary structure, and polarity, based on the AAindex database. We also use this information to encode each amino acid residue for determining the methylation sites in this study.

**Predicted Secondary Structure (SS)**

PSIPRED^[17](#_ENREF_17)^ is a neural-network-based secondary structure prediction tool, which shares a relatively high accuracy. Its outputs have three kinds of secondary structures: H (alpha-helix), E (beta-strand) and C (coil). In this paper, we use a 3-bit binary to encode these three types, i.e. H for 100, E for 001, C for 010.

**Predicted Solvent Accessibility (RSA)**

SPINE-X[^18^](#_ENREF_18) is an accurate multistep neural-network method that can predict secondary structure, solvent accessibility, and backbone torsion angles. In this paper, we use the values of RSA, psi, and phi as features for lysine methylation prediction.

**Predicted Disorder Scores**

DISOPRED2[^19^](#_ENREF_19)^,^[^20^](#_ENREF_20) is proposed for recognizing natively disordered regions based on the amino acid sequence. Based on the predicted values of this method, each residue can be divided into two types: disordered and ordered. In this paper, we use 1-bit binary to encode the feature, i.e. disorder for 1 and order for 0.

**Predicted** **Half Sphere Exposure (HSE)**

Half sphere exposure (HSE) is firstly introduced by Hamelryck[^21^](#_ENREF_21) to measure the solvent exposure of a protein, which plays a fundamentally important role in predicting discontinuous B-cell epitopes[^22^](#_ENREF_22)^,^[^23^](#_ENREF_23). The measure means the number of C_α_ atoms in two half-spheres around a residue's C_α_ atom. On the one hand, HSE can be classified as HSEA and HSEB based on whether only the information about the C_α_ atoms is available. On the other hand, it can also be divided into HSE-up (HSEU) and HSE-down (HSED) depending on the selected C_α_ atoms is in an up half-sphere (U) or a down half-sphere (D). SPIDER-HSE[^24^](#_ENREF_24) is a consistent performance method designed for predicting both HSEA and HSEB of each residue in a certain protein.

**Position-Specific Scoring Matrix (PSSM) by PSI-BLAST**

Position-specific scoring matrix (PSSM) can be calculated from multiple sequence alignment containing the evolutionary information of protein sequences. In this part, the PSSM for each sequence is generated by PSI-BLAST^[25](#_ENREF_25)^ based on the NR database. The PSI-BLAST program can generate two types of position-specific scoring matrices, conservation scores, and probabilities of occurrences. We used both of them to encode for predicting lysine methylation sites.

Additionally, we calculate the evolutionary conservation score, which is defined as:

$$ECS(i)=-\sum_{j=1}^{20} p_{i,j}\log{}_{2}(p_{i,j})$$

where *p_i,j_* represents the probability of amino acid *j* at position *i* of the sliding window (the sliding window size is 2*L*+1).

**Position-Specific Scoring Matrix by HHblits (HH)**

HHblits[^26^](#_ENREF_26) is an open-source, general-purpose tool that can build protein multiple-sequence alignments (MSAs) by profile hidden Markov models (HMMs), which is considered somewhat faster than PSI-BLAST. Here, we employe this novel tool to obtain the probabilities of occurrence, and then calculate the evolutionary conservation scores as mentioned above in prediction.

**Text S3. Structure model-based features**

**RSA**

Solvent accessible surface area (ASA) is calculated by NACCESS[^27^](#_ENREF_27). Relative solvent accessibility (RSA) is defined as the ratio of the ASA of a residue, observed in its three-dimensional structure, to that observed in an extended tri-peptide conformation.

**HSE**

Half-Sphere Exposure (HSE) is a 2D measure, introduced by Hamelryck. HSE consists of the number of C_α_ atoms in two half-spheres around a residue’s C_α_ atom. One of the half-spheres corresponds to the side chain’s neighbourhood, the other half-sphere is in the opposite direction. There are two ways to compute HSE, depending on whether the information is available about both the C_α_ and C_β_ positions (HSEB) or only about the C_α_ positions (HSEA). HSE can be divided into HSEAU, HSEAD, HSEBU, and HSEBD, depending on whether the half-sphere selected is an up half-sphere (U) or a down half-sphere (D).

**Residue Depth**

Chakravarty[^28^](#_ENREF_28) defined the depth of an atom in a protein as the distance between the atom and the nearest surface water molecule. The residue depth is the average of the constituent atom depths.

**L_1_ depth**

The L_1_ depth (L_1_D)[^29^](#_ENREF_29) of a point x with respect to a data set S={X_1,_ X_2…_X_n_} in R^d^ is one minus average of the unit vectors from x to all observations in S.

where η_i_ is a weight assigned to observation X_i_ (and is 1 if all observations are unique), and ||x-X_i_|| is the Euclidean distance between x and X_i_. The time complexity of L_1_ depth is$O(N)$, which is much faster to calculate. Therefore, we calculate the L_1_ depth for each atom of the protein structure instead of each residue.

For a protein structure, the Local L_1_ atom Statistical Depth (L_1_ASD_lR_) is defined as:

$$L_{1}ASD_{lR}(P_{i})=1-\frac{1}{n}|\sum_{j\neq i}^{|p_{j}p_{i}|<R} \frac{\overrightarrow{P_{j}P_{i}}}{|P_{j}P_{i}|}|$$

And local L_1_ residue statistical depth (R) (L_1_RSD_lR_) is the average of L_1_ASD_lR_ for all atoms in a residue, where R represents the local radius. For example, L_1_RSD_l17_ means the distance cutoff of the local L_1_ statistical depth is 17 Å.

**Convex hull of protein surface (CHOPS)**

CHOPS is a special kind of convex hull, which has been proven very useful for identifying B-cell epitope residues[^23^](#_ENREF_23). Atoms locating on protein surface where it is much easy to contact with other proteins will have lower CHOPS score, while atoms locating in pockets of protein surface where it is not easy to contact with other proteins will have higher CHOPS score.

In mathematics, the convex hull of a set X of points in Euclidean space is the smallest convex set that contains X. For instance, when X is a bounded subset of the plane, the convex hull may be visualized as the shape formed by a rubber band stretched around X. For a protein, we consider all atoms in a protein to be a point set X**.** Let X be all the atoms of the exposed residues (RSA>0) in a protein. Atoms that are on the convex hull surface are defined as the first level of the convex hull, denoted as CH_1_(X). The remaining atoms of the protein surface comprise the set X-CH_1_(X). We then compute the convex hull of X-CH_1_(X), such that the atoms on the convex hull surface of X-CH_1_(X) are defined as the secondary level of the convex hull of the protein, denoted as CH_2_(X). Generally speaking, the k-th level of the convex hull of the protein is the convex hull of the set X-∪_i = 1...k-1_{CH_i_(X)}, denoted as CH_k_(X). Finally, one protein surface can represent a union of *n* convex hulls, i.e. X = ∪_i = 1...n_ {CH_i_(X)}. The level of the k-th convex hull of a residue is defined as the minimal level of convex hulls of atoms that are in the residue. For instance, a residue contains C_α_, C_β_, N, O atoms, where C_α_∈CH_2_(X), C_β_∈CH_3_(X), N∈CH_3_(X), and O∈CH_5_(X). The level of the convex hull for this residue is 2, as that is the minimal of {2, 3, 3, 5}.

# **Supporting Tables**

**Table S1.** Statistics of the number of positive and negative samples for lysine and arginine methylation datasets, covering different types of methylation (mono- and di- for arginine, and mono-, di-, and tri- for lysine). *r* is the expected ratio of negative to positive numbers in the real-world data.

| **Residue** | **Dataset** | **Methylation types** | **# negatives** | **# positives** | ***r*** |
| --- | --- | --- | --- | --- | --- |
| K | Training set | mono- | 256 | 465 | 0.551 |
|  |  | di- | 549 | 172 | 3.192 |
|  |  | tri- | 604 | 117 | 5.162 |
|  |  | Total | 18462 | 721 | 25.606 |
|  | Independent test set I | mono- | 70 | 110 | 0.636 |
|  |  | di- | 135 | 45 | 3.000 |
|  |  | tri- | 148 | 32 | 4.625 |
|  |  | Total | 4676 | 180 | 25.978 |
|  | Independent test set II | mono- | 20 | 4973 | 0.004 |
|  |  | di- | 4972 | 21 | 236.762 |
|  |  | tri- | 4987 | 6 | 831.167 |
|  |  | Total | 112322 | 4993 | 22.496 |
| R | Training set | mono- | 395 | 883 | 0.447 |
|  |  | di- | 799 | 479 | 1.668 |
|  |  | Total | 29944 | 1278 | 23.430 |
|  | Independent test set I | mono- | 80 | 231 | 0.346 |
|  |  | di- | 208 | 103 | 2.019 |
|  |  | Total | 7484 | 311 | 24.064 |
|  | Independent test set II | mono- | 3093 | 323 | 9.576 |
|  |  | di- | 3199 | 217 | 14.742 |
|  |  | Total | 88385 | 3416 | 25.874 |

**Table S2.** Statistical test between positive and negative samples on the structure dataset for arginine samples. The Student’s t-test or Wilcoxon signed-rank test is adopted based on whether the samples follow a normal distribution or not by the Shapiro-Wilk test. The pairwise two-tailed test is made to test whether there are significant differences between positives and negatives. If there are significant differences, the pairwise one-tailed test is used to test whether the average value of one group is significantly higher or lower than the average value of the other group.

| **Feature** | **Positive** | **Negative** | **Test Type** | ***P*-value** | **Result** |
| --- | --- | --- | --- | --- | --- |
| CHOPS | 5.984 | 7.615 | one-tailed | P=0.002 | < |
| HSEAD_5_ | 2.188 | 2.262 | two-tailed | P>=0.05 | = |
| HSEAD_6_ | 4.375 | 4.491 | two-tailed | P>=0.05 | = |
| HSEAD_7_ | 5.813 | 6.022 | two-tailed | P>=0.05 | = |
| HSEAD_8_ | 6.617 | 6.743 | two-tailed | P>=0.05 | = |
| HSEAD_9_ | 8.445 | 8.663 | two-tailed | P>=0.05 | = |
| HSEAD_10_ | 10.742 | 10.995 | two-tailed | P>=0.05 | = |
| HSEAD_11_ | 13.430 | 14.018 | two-tailed | P>=0.05 | = |
| HSEAD_12_ | 16.094 | 17.022 | two-tailed | P>=0.05 | = |
| HSEAD_13_ | 19.609 | 20.730 | two-tailed | P>=0.05 | = |
| HSEAD_14_ | 23.203 | 24.769 | two-tailed | P>=0.05 | = |
| HSEAD_15_ | 27.375 | 29.807 | one-tailed | P=0.012 | < |
| HSEAD_16_ | 31.594 | 34.692 | one-tailed | P=0.008 | < |
| HSEAD_17_ | 35.953 | 39.807 | one-tailed | P=0.005 | < |
| HSEAD_18_ | 40.484 | 44.876 | one-tailed | P=0.005 | < |
| HSEAD_19_ | 44.867 | 50.232 | one-tailed | P=0.003 | < |
| HSEAD_20_ | 49.867 | 55.987 | one-tailed | P=0.003 | < |
| HSEAD_21_ | 54.922 | 61.867 | one-tailed | P=0.002 | < |
| HSEAD_22_ | 59.938 | 67.801 | one-tailed | P=0.002 | < |
| HSEAD_23_ | 64.945 | 73.787 | one-tailed | P=0.001 | < |
| HSEAD_24_ | 69.984 | 80.135 | one-tailed | P=0.001 | < |
| HSEAD_25_ | 75.109 | 86.461 | one-tailed | P=0.001 | < |
| HSEAD_26_ | 80.109 | 92.899 | one-tailed | P=0.0004 | < |
| HSEAD_27_ | 84.969 | 99.131 | one-tailed | P=0.0003 | < |
| HSEAD_28_ | 89.414 | 105.290 | one-tailed | P=0.0001 | < |
| HSEAD_29_ | 93.992 | 111.432 | one-tailed | P=9.23e-05 | < |
| HSEAD_30_ | 98.406 | 117.430 | one-tailed | P=6.887e-05 | < |
| HSEAU_5_ | 0.281 | 0.199 | two-tailed | P>=0.05 | = |
| HSEAU_6_ | 1.039 | 0.813 | one-tailed | P=0.019 | > |
| HSEAU_7_ | 1.695 | 1.566 | two-tailed | P>=0.05 | = |
| HSEAU_8_ | 2.523 | 2.357 | two-tailed | P>=0.05 | = |
| HSEAU_9_ | 3.727 | 3.468 | two-tailed | P>=0.05 | = |
| HSEAU_10_ | 5.336 | 5.095 | two-tailed | P>=0.05 | = |
| HSEAU_11_ | 7.375 | 7.215 | two-tailed | P>=0.05 | = |
| HSEAU_12_ | 9.156 | 9.262 | two-tailed | P>=0.05 | = |
| HSEAU_13_ | 11.250 | 11.417 | two-tailed | P>=0.05 | = |
| HSEAU_14_ | 13.484 | 13.826 | two-tailed | P>=0.05 | = |
| HSEAU_15_ | 15.828 | 16.465 | two-tailed | P>=0.05 | = |
| HSEAU_16_ | 17.906 | 19.299 | two-tailed | P>=0.05 | = |
| HSEAU_17_ | 20.648 | 22.237 | two-tailed | P>=0.05 | = |
| HSEAU_18_ | 23.156 | 25.237 | two-tailed | P>=0.05 | = |
| HSEAU_19_ | 25.773 | 28.539 | two-tailed | P>=0.05 | = |
| HSEAU_20_ | 28.852 | 31.997 | two-tailed | P>=0.05 | = |
| HSEAU_21_ | 31.781 | 35.683 | two-tailed | P>=0.05 | = |
| HSEAU_22_ | 34.883 | 39.415 | two-tailed | P>=0.05 | = |
| HSEAU_23_ | 38.023 | 43.312 | one-tailed | P=0.022 | < |
| HSEAU_24_ | 41.477 | 47.235 | one-tailed | P=0.025 | < |
| HSEAU_25_ | 44.836 | 51.222 | one-tailed | P=0.024 | < |
| HSEAU_26_ | 47.750 | 55.233 | one-tailed | P=0.017 | < |
| HSEAU_27_ | 51.109 | 59.179 | one-tailed | P=0.018 | < |
| HSEAU_28_ | 54.266 | 63.143 | one-tailed | P=0.016 | < |
| HSEAU_29_ | 57.609 | 67.100 | one-tailed | P=0.018 | < |
| HSEAU_30_ | 60.883 | 71.044 | one-tailed | P=0.018 | < |
| HSEBD_5_ | 2.281 | 2.243 | two-tailed | P>=0.05 | = |
| HSEBD_6_ | 4.211 | 4.071 | two-tailed | P>=0.05 | = |
| HSEBD_7_ | 5.438 | 5.226 | two-tailed | P>=0.05 | = |
| HSEBD_8_ | 6.359 | 6.003 | one-tailed | P=0.012 | > |
| HSEBD_9_ | 8.188 | 7.733 | one-tailed | P=0.023 | > |
| HSEBD_10_ | 10.531 | 9.906 | one-tailed | P=0.023 | > |
| HSEBD_11_ | 13.195 | 12.752 | two-tailed | P>=0.05 | = |
| HSEBD_12_ | 15.727 | 15.666 | two-tailed | P>=0.05 | = |
| HSEBD_13_ | 19.172 | 19.148 | two-tailed | P>=0.05 | = |
| HSEBD_14_ | 22.859 | 23.073 | two-tailed | P>=0.05 | = |
| HSEBD_15_ | 27.086 | 27.762 | two-tailed | P>=0.05 | = |
| HSEBD_16_ | 31.016 | 32.387 | two-tailed | P>=0.05 | = |
| HSEBD_17_ | 35.594 | 37.240 | two-tailed | P>=0.05 | = |
| HSEBD_18_ | 40.102 | 42.069 | two-tailed | P>=0.05 | = |
| HSEBD_19_ | 44.547 | 47.197 | two-tailed | P>=0.05 | = |
| HSEBD_20_ | 49.648 | 52.651 | two-tailed | P>=0.05 | = |
| HSEBD_21_ | 54.531 | 58.219 | two-tailed | P>=0.05 | = |
| HSEBD_22_ | 59.703 | 63.857 | two-tailed | P>=0.05 | = |
| HSEBD_23_ | 64.828 | 69.588 | two-tailed | P>=0.05 | = |
| HSEBD_24_ | 69.969 | 75.564 | two-tailed | P>=0.05 | = |
| HSEBD_25_ | 75.156 | 81.568 | one-tailed | P=0.023 | < |
| HSEBD_26_ | 80.000 | 87.696 | one-tailed | P=0.013 | < |
| HSEBD_27_ | 84.773 | 93.553 | one-tailed | P=0.009 | < |
| HSEBD_28_ | 89.328 | 99.303 | one-tailed | P=0.006 | < |
| HSEBD_29_ | 93.922 | 105.075 | one-tailed | P=0.004 | < |
| HSEBD_30_ | 98.219 | 110.727 | one-tailed | P=0.003 | < |
| HSEBU_5_ | 0.203 | 0.228 | two-tailed | P>=0.05 | = |
| HSEBU_6_ | 1.219 | 1.249 | two-tailed | P>=0.05 | = |
| HSEBU_7_ | 2.109 | 2.385 | one-tailed | P=0.013 | < |
| HSEBU_8_ | 2.820 | 3.128 | two-tailed | P>=0.05 | = |
| HSEBU_9_ | 4.047 | 4.439 | two-tailed | P>=0.05 | = |
| HSEBU_10_ | 5.633 | 6.246 | one-tailed | P=0.020 | < |
| HSEBU_11_ | 7.742 | 8.554 | two-tailed | P>=0.05 | = |
| HSEBU_12_ | 9.664 | 10.711 | one-tailed | P=0.023 | < |
| HSEBU_13_ | 11.891 | 13.117 | one-tailed | P=0.025 | < |
| HSEBU_14_ | 14.063 | 15.665 | one-tailed | P=0.017 | < |
| HSEBU_15_ | 16.367 | 18.683 | one-tailed | P=0.005 | < |
| HSEBU_16_ | 18.813 | 21.808 | one-tailed | P=0.002 | < |
| HSEBU_17_ | 21.398 | 25.040 | one-tailed | P=0.001 | < |
| HSEBU_18_ | 23.969 | 28.318 | one-tailed | P=0.001 | < |
| HSEBU_19_ | 26.570 | 31.883 | one-tailed | P=0.0004 | < |
| HSEBU_20_ | 29.594 | 35.690 | one-tailed | P=0.0004 | < |
| HSEBU_21_ | 32.773 | 39.735 | one-tailed | P=0.0003 | < |
| HSEBU_22_ | 35.828 | 43.804 | one-tailed | P=0.0003 | < |
| HSEBU_23_ | 38.906 | 48.005 | one-tailed | P=0.0002 | < |
| HSEBU_24_ | 42.375 | 52.349 | one-tailed | P=0.0003 | < |
| HSEBU_25_ | 45.766 | 56.721 | one-tailed | P=0.0003 | < |
| HSEBU_26_ | 48.938 | 61.092 | one-tailed | P=0.0002 | < |
| HSEBU_27_ | 52.516 | 65.481 | one-tailed | P=0.0003 | < |
| HSEBU_28_ | 55.656 | 69.915 | one-tailed | P=0.0002 | < |
| HSEBU_29_ | 59.086 | 74.312 | one-tailed | P=0.0003 | < |
| HSEBU_30_ | 62.617 | 78.657 | one-tailed | P=0.0004 | < |
| L_1_DEPTH_5_ | 0.760 | 0.743 | two-tailed | P>=0.05 | = |
| L_1_DEPTH_6_ | 0.772 | 0.754 | two-tailed | P>=0.05 | = |
| L_1_DEPTH_7_ | 0.774 | 0.758 | two-tailed | P>=0.05 | = |
| L_1_DEPTH_8_ | 0.760 | 0.749 | two-tailed | P>=0.05 | = |
| L_1_DEPTH_9_ | 0.736 | 0.729 | two-tailed | P>=0.05 | = |
| L_1_DEPTH_10_ | 0.711 | 0.706 | two-tailed | P>=0.05 | = |
| L_1_DEPTH_11_ | 0.684 | 0.683 | two-tailed | P>=0.05 | = |
| L_1_DEPTH_12_ | 0.661 | 0.661 | two-tailed | P>=0.05 | = |
| L_1_DEPTH_13_ | 0.637 | 0.642 | two-tailed | P>=0.05 | = |
| L_1_DEPTH_14_ | 0.616 | 0.623 | two-tailed | P>=0.05 | = |
| L_1_DEPTH_15_ | 0.593 | 0.604 | two-tailed | P>=0.05 | = |
| L_1_DEPTH_16_ | 0.575 | 0.589 | two-tailed | P>=0.05 | = |
| L_1_DEPTH_17_ | 0.558 | 0.574 | two-tailed | P>=0.05 | = |
| L_1_DEPTH_18_ | 0.541 | 0.561 | two-tailed | P>=0.05 | = |
| L_1_DEPTH_19_ | 0.525 | 0.548 | two-tailed | P>=0.05 | = |
| L_1_DEPTH_20_ | 0.511 | 0.536 | one-tailed | P=0.025 | < |
| L_1_DEPTH_21_ | 0.498 | 0.525 | one-tailed | P=0.015 | < |
| L_1_DEPTH_22_ | 0.485 | 0.514 | one-tailed | P=0.010 | < |
| L_1_DEPTH_23_ | 0.473 | 0.504 | one-tailed | P=0.006 | < |
| L_1_DEPTH_24_ | 0.462 | 0.494 | one-tailed | P=0.005 | < |
| L_1_DEPTH_25_ | 0.452 | 0.485 | one-tailed | P=0.004 | < |
| L_1_DEPTH_26_ | 0.443 | 0.476 | one-tailed | P=0.003 | < |
| L_1_DEPTH_27_ | 0.434 | 0.468 | one-tailed | P=0.003 | < |
| L_1_DEPTH_28_ | 0.427 | 0.460 | one-tailed | P=0.003 | < |
| L_1_DEPTH_29_ | 0.419 | 0.453 | one-tailed | P=0.003 | < |
| L_1_DEPTH_30_ | 0.413 | 0.446 | one-tailed | P=0.003 | < |
| N1ASA | 22.811 | 22.540 | two-tailed | P>=0.05 | = |
| N1CHOPS | 7.813 | 9.633 | one-tailed | P=0.003 | < |
| N1L_1_DEPTH_5_ | 0.557 | 0.555 | two-tailed | P>=0.05 | = |
| N1L_1_DEPTH_6_ | 0.547 | 0.546 | two-tailed | P>=0.05 | = |
| N1L_1_DEPTH_7_ | 0.536 | 0.540 | two-tailed | P>=0.05 | = |
| N1L_1_DEPTH_8_ | 0.529 | 0.532 | two-tailed | P>=0.05 | = |
| N1L_1_DEPTH_9_ | 0.523 | 0.528 | two-tailed | P>=0.05 | = |
| N1L_1_DEPTH_10_ | 0.520 | 0.525 | two-tailed | P>=0.05 | = |
| N1L_1_DEPTH_11_ | 0.515 | 0.523 | two-tailed | P>=0.05 | = |
| N1L_1_DEPTH_12_ | 0.508 | 0.519 | two-tailed | P>=0.05 | = |
| N1L_1_DEPTH_13_ | 0.499 | 0.513 | two-tailed | P>=0.05 | = |
| N1L_1_DEPTH_14_ | 0.491 | 0.507 | two-tailed | P>=0.05 | = |
| N1L_1_DEPTH_15_ | 0.481 | 0.500 | two-tailed | P>=0.05 | = |
| N1L_1_DEPTH_16_ | 0.471 | 0.492 | two-tailed | P>=0.05 | = |
| N1L_1_DEPTH_17_ | 0.460 | 0.484 | two-tailed | P>=0.05 | = |
| N1L_1_DEPTH_18_ | 0.450 | 0.475 | two-tailed | P>=0.05 | = |
| N1L_1_DEPTH_19_ | 0.440 | 0.467 | two-tailed | P>=0.05 | = |
| N1L_1_DEPTH_20_ | 0.430 | 0.460 | one-tailed | P=0.019 | < |
| N1L_1_DEPTH_21_ | 0.420 | 0.452 | one-tailed | P=0.012 | < |
| N1L_1_DEPTH_22_ | 0.412 | 0.445 | one-tailed | P=0.010 | < |
| N1L_1_DEPTH_23_ | 0.405 | 0.438 | one-tailed | P=0.008 | < |
| N1L_1_DEPTH_24_ | 0.397 | 0.432 | one-tailed | P=0.006 | < |
| N1L_1_DEPTH_25_ | 0.390 | 0.426 | one-tailed | P=0.005 | < |
| N1L_1_DEPTH_26_ | 0.384 | 0.419 | one-tailed | P=0.005 | < |
| N1L_1_DEPTH_27_ | 0.377 | 0.413 | one-tailed | P=0.004 | < |
| N1L_1_DEPTH_28_ | 0.371 | 0.408 | one-tailed | P=0.003 | < |
| N1L_1_DEPTH_29_ | 0.365 | 0.402 | one-tailed | P=0.003 | < |
| N1L_1_DEPTH_30_ | 0.360 | 0.397 | one-tailed | P=0.003 | < |
| N1ResideuDepth | 3.741 | 3.949 | one-tailed | P=0.016 | < |
| N2ASA | 28.567 | 27.375 | two-tailed | P>=0.05 | = |
| N2CHOPS | 7.508 | 9.401 | one-tailed | P=0.002 | < |
| N2L_1_DEPTH_5_ | 0.506 | 0.517 | two-tailed | P>=0.05 | = |
| N2L_1_DEPTH_6_ | 0.506 | 0.519 | two-tailed | P>=0.05 | = |
| N2L_1_DEPTH_7_ | 0.495 | 0.517 | two-tailed | P>=0.05 | = |
| N2L_1_DEPTH_8_ | 0.496 | 0.514 | two-tailed | P>=0.05 | = |
| N2L_1_DEPTH_9_ | 0.497 | 0.512 | two-tailed | P>=0.05 | = |
| N2L_1_DEPTH_10_ | 0.497 | 0.510 | two-tailed | P>=0.05 | = |
| N2L_1_DEPTH_11_ | 0.496 | 0.509 | two-tailed | P>=0.05 | = |
| N2L_1_DEPTH_12_ | 0.492 | 0.506 | two-tailed | P>=0.05 | = |
| N2L_1_DEPTH_13_ | 0.487 | 0.502 | two-tailed | P>=0.05 | = |
| N2L_1_DEPTH_14_ | 0.478 | 0.497 | two-tailed | P>=0.05 | = |
| N2L_1_DEPTH_15_ | 0.470 | 0.490 | two-tailed | P>=0.05 | = |
| N2L_1_DEPTH_16_ | 0.461 | 0.483 | two-tailed | P>=0.05 | = |
| N2L_1_DEPTH_17_ | 0.454 | 0.476 | two-tailed | P>=0.05 | = |
| N2L_1_DEPTH_18_ | 0.444 | 0.468 | two-tailed | P>=0.05 | = |
| N2L_1_DEPTH_19_ | 0.434 | 0.461 | two-tailed | P>=0.05 | = |
| N2L_1_DEPTH_20_ | 0.425 | 0.454 | one-tailed | P=0.024 | < |
| N2L_1_DEPTH_21_ | 0.416 | 0.447 | one-tailed | P=0.016 | < |
| N2L_1_DEPTH_22_ | 0.408 | 0.440 | one-tailed | P=0.012 | < |
| N2L_1_DEPTH_23_ | 0.400 | 0.433 | one-tailed | P=0.010 | < |
| N2L_1_DEPTH_24_ | 0.394 | 0.427 | one-tailed | P=0.008 | < |
| N2L_1_DEPTH_25_ | 0.387 | 0.421 | one-tailed | P=0.007 | < |
| N2L_1_DEPTH_26_ | 0.380 | 0.415 | one-tailed | P=0.005 | < |
| N2L_1_DEPTH_27_ | 0.375 | 0.409 | one-tailed | P=0.006 | < |
| N2L_1_DEPTH_28_ | 0.368 | 0.404 | one-tailed | P=0.004 | < |
| N2L_1_DEPTH_29_ | 0.363 | 0.398 | one-tailed | P=0.004 | < |
| N2L_1_DEPTH_30_ | 0.358 | 0.393 | one-tailed | P=0.004 | < |
| N2ResidueDepth | 3.651 | 3.864 | one-tailed | P=0.010 | < |
| ResidueDepth | 4.432 | 4.657 | one-tailed | P=0.008 | < |
| RSA | 0.431 | 0.419 | two-tailed | P>=0.05 | = |
| SideChainRSA | 0.473 | 0.460 | two-tailed | P>=0.05 | = |

**Table S3.** Statistical test between positive and negative samples on the structure dataset for lysine samples. The Student’s t-test or Wilcoxon signed-rank test is adopted based on whether the samples follow a normal distribution or not by the Shapiro-Wilk test. The pairwise two-tailed test is made to test whether there are significant differences between positives and negatives. If there are significant differences, the pairwise one-tailed test is used to test whether the average value of one group is significantly higher or lower than the average value of the other group.

| **Feature** | **Positive** | **Negative** | **Test Type** | ***P*-value** | **Result** |
| --- | --- | --- | --- | --- | --- |
| CHOPS | 4.399 | 6.759 | one-tailed | P=4.57e-08 | < |
| HSEAD_5_ | 2.161 | 2.223 | two-tailed | P>=0.05 | = |
| HSEAD_6_ | 4.294 | 4.416 | two-tailed | P>=0.05 | = |
| HSEAD_7_ | 5.679 | 5.888 | two-tailed | P>=0.05 | = |
| HSEAD_8_ | 6.376 | 6.614 | two-tailed | P>=0.05 | = |
| HSEAD_9_ | 8.128 | 8.505 | one-tailed | P=0.014 | < |
| HSEAD_10_ | 10.312 | 10.805 | one-tailed | P=0.014 | < |
| HSEAD_11_ | 12.890 | 13.755 | one-tailed | P=0.001 | < |
| HSEAD_12_ | 15.408 | 16.621 | one-tailed | P=0.0005 | < |
| HSEAD_13_ | 18.587 | 20.360 | one-tailed | P=6.52e-05 | < |
| HSEAD_14_ | 22.069 | 24.573 | one-tailed | P=1.29e-05 | < |
| HSEAD_15_ | 26.376 | 29.442 | one-tailed | P=2.80e-05 | < |
| HSEAD_16_ | 30.234 | 34.405 | one-tailed | P=1.43e-06 | < |
| HSEAD_17_ | 34.477 | 39.432 | one-tailed | P=1.07e-06 | < |
| HSEAD_18_ | 38.839 | 44.584 | one-tailed | P=1.05e-06 | < |
| HSEAD_19_ | 43.619 | 50.135 | one-tailed | P=1.53e-06 | < |
| HSEAD_20_ | 48.619 | 55.931 | one-tailed | P=2.275e-06 | < |
| HSEAD_21_ | 53.440 | 62.012 | one-tailed | P=9.13e-07 | < |
| HSEAD_22_ | 58.638 | 68.200 | one-tailed | P=1.06e-06 | < |
| HSEAD_23_ | 63.940 | 74.711 | one-tailed | P=9.241e-07 | < |
| HSEAD_24_ | 69.239 | 81.409 | one-tailed | P=5.94e-07 | < |
| HSEAD_25_ | 74.688 | 88.346 | one-tailed | P=3.95e-07 | < |
| HSEAD_26_ | 80.096 | 95.257 | one-tailed | P=2.58e-07 | < |
| HSEAD_27_ | 85.628 | 102.244 | one-tailed | P=2.35e-07 | < |
| HSEAD_28_ | 90.858 | 109.330 | one-tailed | P=1.16e-07 | < |
| HSEAD_29_ | 96.477 | 116.458 | one-tailed | P=1.181e-07 | < |
| HSEAD_30_ | 101.839 | 123.498 | one-tailed | P=9.039e-08 | < |
| HSEAU_5_ | 0.147 | 0.164 | two-tailed | P>=0.05 | = |
| HSEAU_6_ | 0.619 | 0.679 | two-tailed | P>=0.05 | = |
| HSEAU_7_ | 1.216 | 1.304 | two-tailed | P>=0.05 | = |
| HSEAU_8_ | 1.761 | 1.925 | two-tailed | P>=0.05 | = |
| HSEAU_9_ | 2.450 | 2.856 | one-tailed | P=0.007 | < |
| HSEAU_10_ | 3.679 | 4.182 | one-tailed | P=0.017 | < |
| HSEAU_11_ | 5.046 | 5.894 | one-tailed | P=0.003 | < |
| HSEAU_12_ | 6.422 | 7.563 | one-tailed | P=0.002 | < |
| HSEAU_13_ | 7.839 | 9.389 | one-tailed | P=0.0006 | < |
| HSEAU_14_ | 9.440 | 11.372 | one-tailed | P=0.0004 | < |
| HSEAU_15_ | 11.248 | 13.583 | one-tailed | P=0.0004 | < |
| HSEAU_16_ | 13.161 | 15.885 | one-tailed | P=0.0004 | < |
| HSEAU_17_ | 15.321 | 18.441 | one-tailed | P=0.0005 | < |
| HSEAU_18_ | 17.335 | 21.060 | one-tailed | P=0.0003 | < |
| HSEAU_19_ | 19.459 | 23.921 | one-tailed | P=0.0002 | < |
| HSEAU_20_ | 21.899 | 26.902 | one-tailed | P=0.0002 | < |
| HSEAU_21_ | 24.358 | 30.087 | one-tailed | P=0.0002 | < |
| HSEAU_22_ | 26.826 | 33.287 | one-tailed | P=0.0001 | < |
| HSEAU_23_ | 29.372 | 36.619 | one-tailed | P=0.0001 | < |
| HSEAU_24_ | 31.904 | 40.078 | one-tailed | P=9.38e-05 | < |
| HSEAU_25_ | 34.528 | 43.578 | one-tailed | P=9.44e-05 | < |
| HSEAU_26_ | 37.234 | 47.179 | one-tailed | P=9.20e-05 | < |
| HSEAU_27_ | 39.913 | 50.795 | one-tailed | P=7.75e-05 | < |
| HSEAU_28_ | 42.482 | 54.500 | one-tailed | P=6.13e-05 | < |
| HSEAU_29_ | 45.317 | 58.230 | one-tailed | P=6.85e-05 | < |
| HSEAU_30_ | 47.931 | 61.984 | one-tailed | P=5.48e-05 | < |
| HSEBD_5_ | 2.174 | 2.237 | one-tailed | P=0.024 | < |
| HSEBD_6_ | 3.881 | 4.011 | two-tailed | P>=0.05 | = |
| HSEBD_7_ | 5.041 | 5.177 | two-tailed | P>=0.05 | = |
| HSEBD_8_ | 5.798 | 5.933 | two-tailed | P>=0.05 | = |
| HSEBD_9_ | 7.335 | 7.665 | one-tailed | P=0.020 | < |
| HSEBD_10_ | 9.486 | 9.836 | two-tailed | P>=0.05 | = |
| HSEBD_11_ | 11.839 | 12.539 | one-tailed | P=0.008 | < |
| HSEBD_12_ | 14.289 | 15.278 | one-tailed | P=0.005 | < |
| HSEBD_13_ | 17.326 | 18.770 | one-tailed | P=0.001 | < |
| HSEBD_14_ | 20.670 | 22.838 | one-tailed | P=0.0002 | < |
| HSEBD_15_ | 24.743 | 27.362 | one-tailed | P=0.0003 | < |
| HSEBD_16_ | 28.509 | 32.021 | one-tailed | P=5.02e-05 | < |
| HSEBD_17_ | 32.596 | 36.862 | one-tailed | P=2.77e-05 | < |
| HSEBD_18_ | 36.771 | 41.794 | one-tailed | P=1.88e-05 | < |
| HSEBD_19_ | 41.367 | 47.102 | one-tailed | P=2.39e-05 | < |
| HSEBD_20_ | 46.142 | 52.621 | one-tailed | P=2.75e-05 | < |
| HSEBD_21_ | 50.752 | 58.365 | one-tailed | P=1.13e-05 | < |
| HSEBD_22_ | 55.583 | 64.286 | one-tailed | P=7.19e-06 | < |
| HSEBD_23_ | 60.578 | 70.469 | one-tailed | P=5.16e-06 | < |
| HSEBD_24_ | 65.716 | 76.820 | one-tailed | P=4.12e-06 | < |
| HSEBD_25_ | 70.775 | 83.373 | one-tailed | P=1.99e-06 | < |
| HSEBD_26_ | 75.982 | 89.949 | one-tailed | P=1.38e-06 | < |
| HSEBD_27_ | 81.151 | 96.557 | one-tailed | P=1.10e-06 | < |
| HSEBD_28_ | 86.046 | 103.258 | one-tailed | P=5.01e-07 | < |
| HSEBD_29_ | 91.394 | 110.054 | one-tailed | P=4.91e-07 | < |
| HSEBD_30_ | 96.459 | 116.701 | one-tailed | P=3.95e-07 | < |
| HSEBU_5_ | 0.156 | 0.166 | two-tailed | P>=0.05 | = |
| HSEBU_6_ | 1.092 | 1.115 | two-tailed | P>=0.05 | = |
| HSEBU_7_ | 1.936 | 2.059 | two-tailed | P>=0.05 | = |
| HSEBU_8_ | 2.427 | 2.660 | two-tailed | P>=0.05 | = |
| HSEBU_9_ | 3.353 | 3.771 | one-tailed | P=0.005 | < |
| HSEBU_10_ | 4.661 | 5.251 | one-tailed | P=0.006 | < |
| HSEBU_11_ | 6.284 | 7.243 | one-tailed | P=0.001 | < |
| HSEBU_12_ | 7.775 | 9.069 | one-tailed | P=0.0004 | < |
| HSEBU_13_ | 9.408 | 11.177 | one-tailed | P=8.58e-05 | < |
| HSEBU_14_ | 11.165 | 13.345 | one-tailed | P=5.04e-05 | < |
| HSEBU_15_ | 13.261 | 15.951 | one-tailed | P=4.73e-05 | < |
| HSEBU_16_ | 15.344 | 18.609 | one-tailed | P=3.129e-05 | < |
| HSEBU_17_ | 17.720 | 21.402 | one-tailed | P=4.93e-05 | < |
| HSEBU_18_ | 19.954 | 24.305 | one-tailed | P=3.79e-05 | < |
| HSEBU_19_ | 22.312 | 27.478 | one-tailed | P=1.56e-05 | < |
| HSEBU_20_ | 25.055 | 30.805 | one-tailed | P=2.11e-05 | < |
| HSEBU_21_ | 27.794 | 34.402 | one-tailed | P=1.50e-05 | < |
| HSEBU_22_ | 30.716 | 37.953 | one-tailed | P=2.31e-05 | < |
| HSEBU_23_ | 33.628 | 41.697 | one-tailed | P=2.53e-05 | < |
| HSEBU_24_ | 36.381 | 45.593 | one-tailed | P=1.24e-05 | < |
| HSEBU_25_ | 39.500 | 49.566 | one-tailed | P=1.67e-05 | < |
| HSEBU_26_ | 42.468 | 53.595 | one-tailed | P=1.47e-05 | < |
| HSEBU_27_ | 45.541 | 57.681 | one-tailed | P=1.38e-05 | < |
| HSEBU_28_ | 48.514 | 61.864 | one-tailed | P=1.10e-05 | < |
| HSEBU_29_ | 51.679 | 66.019 | one-tailed | P=1.30e-05 | < |
| HSEBU_30_ | 54.651 | 70.265 | one-tailed | P=9.85e-06 | < |
| L_1_DEPTH_5_ | 0.712 | 0.720 | two-tailed | P>=0.05 | = |
| L_1_DEPTH_6_ | 0.708 | 0.722 | one-tailed | P=0.020 | < |
| L_1_DEPTH_7_ | 0.697 | 0.718 | one-tailed | P=0.003 | < |
| L_1_DEPTH_8_ | 0.682 | 0.706 | one-tailed | P=0.001 | < |
| L_1_DEPTH_9_ | 0.656 | 0.685 | one-tailed | P=0.0005 | < |
| L_1_DEPTH_10_ | 0.631 | 0.661 | one-tailed | P=0.0005 | < |
| L_1_DEPTH_11_ | 0.608 | 0.639 | one-tailed | P=0.0004 | < |
| L_1_DEPTH_12_ | 0.588 | 0.619 | one-tailed | P=0.0004 | < |
| L_1_DEPTH_13_ | 0.569 | 0.600 | one-tailed | P=0.0004 | < |
| L_1_DEPTH_14_ | 0.551 | 0.583 | one-tailed | P=0.0003 | < |
| L_1_DEPTH_15_ | 0.534 | 0.567 | one-tailed | P=0.0002 | < |
| L_1_DEPTH_16_ | 0.518 | 0.552 | one-tailed | P=0.0002 | < |
| L_1_DEPTH_17_ | 0.504 | 0.540 | one-tailed | P=0.0001 | < |
| L_1_DEPTH_18_ | 0.492 | 0.528 | one-tailed | P=8.846e-05 | < |
| L_1_DEPTH_19_ | 0.479 | 0.517 | one-tailed | P=6.81e-05 | < |
| L_1_DEPTH_20_ | 0.468 | 0.506 | one-tailed | P=4.97e-05 | < |
| L_1_DEPTH_21_ | 0.458 | 0.497 | one-tailed | P=3.86e-05 | < |
| L_1_DEPTH_22_ | 0.448 | 0.488 | one-tailed | P=3.33e-05 | < |
| L_1_DEPTH_23_ | 0.438 | 0.479 | one-tailed | P=1.85e-05 | < |
| L_1_DEPTH_24_ | 0.430 | 0.471 | one-tailed | P=2.20e-05 | < |
| L_1_DEPTH_25_ | 0.422 | 0.463 | one-tailed | P=2.16e-05 | < |
| L_1_DEPTH_26_ | 0.415 | 0.456 | one-tailed | P=2.30e-05 | < |
| L_1_DEPTH_27_ | 0.408 | 0.449 | one-tailed | P=2.66e-05 | < |
| L_1_DEPTH_28_ | 0.403 | 0.442 | one-tailed | P=4.64e-05 | < |
| L_1_DEPTH_29_ | 0.397 | 0.436 | one-tailed | P=6.56e-05 | < |
| L_1_DEPTH_30_ | 0.390 | 0.429 | one-tailed | P=6.39e-05 | < |
| NASA | 34.165 | 31.603 | one-tailed | P=0.004 | > |
| NCHOPS | 5.060 | 7.691 | one-tailed | P=4.95e-08 | < |
| NL_1_DEPTH_5_ | 0.398 | 0.423 | one-tailed | P=0.01 | < |
| NL_1_DEPTH_6_ | 0.378 | 0.412 | one-tailed | P=0.0009 | < |
| NL_1_DEPTH_7_ | 0.370 | 0.408 | one-tailed | P=0.0009 | < |
| NL_1_DEPTH_8_ | 0.377 | 0.414 | one-tailed | P=0.002 | < |
| NL_1_DEPTH_9_ | 0.381 | 0.420 | one-tailed | P=0.001 | < |
| NL_1_DEPTH_10_ | 0.384 | 0.424 | one-tailed | P=0.0007 | < |
| NL_1_DEPTH_11_ | 0.388 | 0.428 | one-tailed | P=0.0007 | < |
| NL_1_DEPTH_12_ | 0.390 | 0.431 | one-tailed | P=0.0004 | < |
| NL_1_DEPTH_13_ | 0.390 | 0.431 | one-tailed | P=0.0002 | < |
| NL_1_DEPTH_14_ | 0.389 | 0.430 | one-tailed | P=0.0003 | < |
| NL_1_DEPTH_15_ | 0.386 | 0.427 | one-tailed | P=0.0002 | < |
| NL_1_DEPTH_16_ | 0.381 | 0.423 | one-tailed | P=0.0001 | < |
| NL_1_DEPTH_17_ | 0.377 | 0.419 | one-tailed | P=8.52e-05 | < |
| NL_1_DEPTH_18_ | 0.373 | 0.415 | one-tailed | P=7.70e-05 | < |
| NL_1_DEPTH_19_ | 0.368 | 0.410 | one-tailed | P=6.37e-05 | < |
| NL_1_DEPTH_20_ | 0.362 | 0.406 | one-tailed | P=3.83e-05 | < |
| NL_1_DEPTH_21_ | 0.357 | 0.401 | one-tailed | P=3.29e-05 | < |
| NL_1_DEPTH_22_ | 0.353 | 0.397 | one-tailed | P=2.65e-05 | < |
| NL_1_DEPTH_23_ | 0.348 | 0.392 | one-tailed | P=1.736e-05 | < |
| NL_1_DEPTH_24_ | 0.344 | 0.388 | one-tailed | P=1.93e-05 | < |
| NL_1_DEPTH_25_ | 0.339 | 0.384 | one-tailed | P=1.57e-05 | < |
| NL_1_DEPTH_26_ | 0.335 | 0.380 | one-tailed | P=1.25e-05 | < |
| NL_1_DEPTH_27_ | 0.331 | 0.376 | one-tailed | P=1.39e-05 | < |
| NL_1_DEPTH_28_ | 0.327 | 0.372 | one-tailed | P=1.26e-05 | < |
| NL_1_DEPTH_29_ | 0.323 | 0.367 | one-tailed | P=1.39e-05 | < |
| NL_1_DEPTH_30_ | 0.319 | 0.364 | one-tailed | P=1.48e-05 | < |
| NResidueDepth | 3.344 | 3.424 | two-tailed | P>=0.05 | = |
| ResidueDepth | 4.213 | 4.407 | one-tailed | P=0.020 | < |
| RSA | 0.552 | 0.513 | one-tailed | P=0.005 | > |
| SideChainRSA | 0.624 | 0.578 | one-tailed | P=0.003 | > |

**Table S4.** The comparison of the support vector machine (SVM), neural network (NN), random forest (RF) and Bayes classifiers with different sliding window sizes based on both sequence-based and structure model-based features for lysine (K) methylation prediction on the training set. The accuracy is used as a measure to evaluate the performance of different classifiers.

| **Type** | **Methods** | **SVM** | **NN** | **RF** | **Bayes** |
| --- | --- | --- | --- | --- | --- |
|  | **Window size** |  |  |  |  |
| **K** | 9 | 0.548 | 0.426 | 0.348 | 0.542 |
|  | 11 | 0.519 | 0.493 | 0.354 | 0.551 |
|  | 13 | 0.547 | 0.447 | 0.343 | 0.545 |
|  | 15 | 0.544 | 0.591 | 0.352 | 0.554 |
|  | 17 | 0.555 | 0.336 | 0.346 | 0.556 |
|  | 19 | 0.548 | 0.607 | 0.338 | 0.560 |
|  | 21 | 0.556 | 0.568 | 0.335 | 0.557 |
|  | 23 | 0.540 | 0.497 | 0.377 | 0.556 |
| **K MONO** | 9 | 0.564 | 0.488 | 0.571 | 0.559 |
|  | 11 | 0.587 | 0.498 | 0.564 | 0.549 |
|  | 13 | 0.570 | 0.607 | 0.576 | 0.524 |
|  | 15 | 0.645 | 0.601 | 0.592 | 0.527 |
|  | 17 | 0.534 | 0.613 | 0.562 | 0.526 |
|  | 19 | 0.520 | 0.527 | 0.596 | 0.537 |
|  | 21 | 0.566 | 0.570 | 0.578 | 0.531 |
|  | 23 | 0.564 | 0.619 | 0.564 | 0.524 |
| **K DI** | 9 | 0.639 | 0.656 | 0.748 | 0.606 |
|  | 11 | 0.669 | 0.465 | 0.739 | 0.589 |
|  | 13 | 0.680 | 0.527 | 0.736 | 0.570 |
|  | 15 | 0.648 | 0.656 | 0.743 | 0.577 |
|  | 17 | 0.662 | 0.441 | 0.738 | 0.563 |
|  | 19 | 0.675 | 0.664 | 0.745 | 0.555 |
|  | 21 | 0.666 | 0.621 | 0.743 | 0.563 |
|  | 23 | 0.673 | 0.619 | 0.741 | 0.573 |
| **K TRI** | 9 | 0.741 | 0.782 | 0.829 | 0.506 |
|  | 11 | 0.707 | 0.684 | 0.834 | 0.530 |
|  | 13 | 0.748 | 0.828 | 0.831 | 0.533 |
|  | 15 | 0.723 | 0.628 | 0.832 | 0.553 |
|  | 17 | 0.677 | 0.664 | 0.827 | 0.549 |
|  | 19 | 0.721 | 0.825 | 0.831 | 0.556 |
|  | 21 | 0.723 | 0.649 | 0.835 | 0.574 |
|  | 23 | 0.727 | 0.836 | 0.842 | 0.587 |

**Table S5.** The comparison of the support vector machine (SVM), neural network (NN), random forest (RF) and Bayes classifiers with different sliding window sizes based on both sequence-based and structure model-based features for arginine (R) methylation prediction on the training set. The accuracy is used as a measure to evaluate the performance of different classifiers.

| **Type** | **Methods** | **SVM** | **NN** | **RF** | **BAYES** |
| --- | --- | --- | --- | --- | --- |
|  | **Window size** |  |  |  |  |
| **R** | 9 | 0.608 | 0.414 | 0.468 | 0.630 |
|  | 11 | 0.606 | 0.375 | 0.477 | 0.632 |
|  | 13 | 0.603 | 0.586 | 0.472 | 0.623 |
|  | 15 | 0.608 | 0.538 | 0.475 | 0.623 |
|  | 17 | 0.629 | 0.610 | 0.482 | 0.625 |
|  | 19 | 0.614 | 0.439 | 0.488 | 0.624 |
|  | 21 | 0.603 | 0.474 | 0.470 | 0.625 |
|  | 23 | 0.619 | 0.372 | 0.491 | 0.626 |
| **R MONO** | 9 | 0.657 | 0.603 | 0.631 | 0.586 |
|  | 11 | 0.645 | 0.572 | 0.628 | 0.578 |
|  | 13 | 0.641 | 0.559 | 0.648 | 0.576 |
|  | 15 | 0.646 | 0.679 | 0.638 | 0.567 |
|  | 17 | 0.649 | 0.565 | 0.656 | 0.560 |
|  | 19 | 0.638 | 0.495 | 0.665 | 0.566 |
|  | 21 | 0.653 | 0.584 | 0.649 | 0.563 |
|  | 23 | 0.649 | 0.613 | 0.663 | 0.568 |
| **R DI** | 9 | 0.603 | 0.557 | 0.601 | 0.557 |
|  | 11 | 0.589 | 0.519 | 0.595 | 0.570 |
|  | 13 | 0.585 | 0.549 | 0.605 | 0.557 |
|  | 15 | 0.578 | 0.524 | 0.630 | 0.560 |
|  | 17 | 0.604 | 0.579 | 0.610 | 0.548 |
|  | 19 | 0.597 | 0.518 | 0.603 | 0.549 |
|  | 21 | 0.583 | 0.531 | 0.628 | 0.546 |
|  | 23 | 0.608 | 0.605 | 0.633 | 0.547 |

**Table S6.** The dimensions of feature selection for the Met-predictor based on both sequence-based features and structure model-based features. The value before the “/” means the number of selected features in each type of feature set, while the value after the “/” represents the number of all features in each type of feature set.

| **Feature type** | **K**  **(win=17)** | **K MONO**  **(win=23)** | **K DI**  **(win=15)** | **K TRI**  **(win=23)** | **R**  **(win=17)** | **R MONO**  **(win=17)** | **R DI**  **(win=21)** |
| --- | --- | --- | --- | --- | --- | --- | --- |
| **LC** | 1/3 | 1/3 | 1/3 | 3/3 | 1/3 | 3/3 | 1/3 |
| **PWAA** | 2/20 | 1/20 | 4/20 | 7/20 | 5/20 | 20/20 | 1/20 |
| **EBGW** | 6/15 | 1/15 | 4/15 | 7/15 | 7/15 | 15/15 | 1/15 |
| **CKSAAP** | 48/400 | 1/400 | 73/400 | 66/400 | 1/400 | 400/400 | 1/400 |
| **KNN** | 4/5 | 1/5 | 1/5 | 3/5 | 4/5 | 5/5 | 1/5 |
| **AAindex** | 21/1147 | 13/1177 | 26/1137 | 55/1177 | 26/1147 | 23/1147 | 16/1167 |
| **SS** | 4/108 | 1/144 | 15/96 | 21/144 | 5/108 | 108/108 | 1/132 |
| **seqRSA** | 4/123 | 9/141 | 8/117 | 10/141 | 23/123 | 13/123 | 6/135 |
| **Disorder** | 5/30 | 1/36 | 8/28 | 9/36 | 1/30 | 30/30 | 1/34 |
| **seqHSE** | 1/68 | 4/92 | 5/60 | 3/92 | 7/68 | 2/68 | 2/84 |
| **PSSM** | 22/905 | 12/1175 | 28/815 | 25/1175 | 14/905 | 30/905 | 27/1085 |
| **HH** | 18/357 | 3/483 | 70/315 | 78/483 | 34/357 | 357/357 | 1/441 |
| **CHOPS** | 1/20 | 5/26 | 3/18 | 3/26 | 1/20 | 20/20 | 1/24 |
| **strHSE** | 8/1768 | 1/2392 | 36/1560 | 76/2392 | 17/1768 | 1768/1768 | 3/2184 |
| **Depth** | 8/27 | 8/27 | 2/27 | 2/27 | 8/27 | 27/27 | 8/27 |
| **strRSA** | 1/17 | 1/23 | 1/15 | 1/23 | 1/17 | 17/17 | 1/21 |
| **Total** | 154/5013 | 63/6159 | 285/4631 | 369/6159 | 155/5013 | 2838/5013 | 72/5777 |

**Table S7.** The performance of the feature selection method with different sliding window sizes based on both sequence-based and structure model-based features for lysine (K) methylation prediction on the training set. The accuracy is used as a measure to evaluate the performance of different classifiers. “No” means all of the features are used to build model, while “Yes” means the only features selected by our feature selection method are used.

| **Type** | **Methods** | **No** | **Yes** |
| --- | --- | --- | --- |
|  | **Window size** |  |  |
| **K** | 9 | 0.548 | **0.617** |
|  | 11 | 0.519 | **0.632** |
|  | 13 | 0.547 | **0.594** |
|  | 15 | 0.544 | **0.620** |
|  | 17 | 0.555 | **0.643** |
|  | 19 | 0.548 | **0.628** |
|  | 21 | 0.556 | **0.641** |
|  | 23 | 0.540 | **0.638** |
| **K MONO** | 9 | 0.564 | **0.655** |
|  | 11 | 0.587 | **0.666** |
|  | 13 | 0.570 | **0.644** |
|  | 15 | 0.645 | **0.652** |
|  | 17 | 0.534 | **0.655** |
|  | 19 | 0.520 | **0.652** |
|  | 21 | 0.566 | **0.659** |
|  | 23 | 0.564 | **0.674** |
| **K DI** | 9 | 0.639 | **0.663** |
|  | 11 | **0.669** | 0.657 |
|  | 13 | **0.680** | 0.628 |
|  | 15 | 0.648 | **0.695** |
|  | 17 | 0.662 | **0.683** |
|  | 19 | **0.675** | 0.570 |
|  | 21 | 0.666 | **0.692** |
|  | 23 | 0.673 | **0.677** |
| **K TRI** | 9 | **0.741** | 0.581 |
|  | 11 | **0.707** | 0.628 |
|  | 13 | **0.748** | 0.675 |
|  | 15 | **0.723** | 0.526 |
|  | 17 | **0.677** | 0.568 |
|  | 19 | **0.721** | 0.628 |
|  | 21 | **0.723** | 0.615 |
|  | 23 | **0.727** | 0.679 |

**Table S8.** The performance of the feature selection method with different sliding window sizes based on both sequence-based and structure model-based features for arginine (R) methylation prediction on the training set. The accuracy is used as a measure to evaluate the performance of different classifiers. “No” means all of the features are used to build model, while “Yes” means the only features selected by our feature selection method are used.

| **Type** | **Methods** | **No** | **Yes** |
| --- | --- | --- | --- |
|  | **Window size** |  |  |
| **R** | 9 | 0.608 | **0.671** |
|  | 11 | 0.606 | **0.673** |
|  | 13 | 0.603 | **0.676** |
|  | 15 | 0.608 | **0.687** |
|  | 17 | 0.629 | **0.689** |
|  | 19 | 0.614 | **0.687** |
|  | 21 | 0.603 | **0.673** |
|  | 23 | 0.619 | **0.673** |
| **R MONO** | 9 | 0.657 | **0.688** |
|  | 11 | 0.645 | **0.689** |
|  | 13 | 0.641 | **0.689** |
|  | 15 | 0.646 | **0.690** |
|  | 17 | 0.649 | **0.709** |
|  | 19 | 0.638 | **0.692** |
|  | 21 | 0.653 | **0.689** |
|  | 23 | 0.649 | **0.692** |
| **R DI** | 9 | 0.603 | **0.654** |
|  | 11 | 0.589 | **0.638** |
|  | 13 | 0.585 | **0.661** |
|  | 15 | 0.578 | **0.656** |
|  | 17 | 0.604 | **0.653** |
|  | 19 | 0.597 | **0.656** |
|  | 21 | 0.583 | **0.692** |
|  | 23 | 0.608 | **0.685** |

**Table S9.** The prediction accuracy (ACC) of different models based on 16 subtypes of features, all sequence-based features, and all sequence-based and structure model-based features for lysine (K) and arginine (R). The bar labelled “All seq” corresponds to the accuracy of model built by only sequence-based features, while the bar labelled “All seq+str” represents the accuracy of model built by both sequence-based and structure model-based features. This table gives the accuracy values of Figure 6A.

| **Feature type** | **K** | **R** |
| --- | --- | --- |
| **LC** | 0.4889 | 0.4968 |
| **PWAA** | 0.4917 | 0.4920 |
| **EBGW** | 0.5583 | 0.6447 |
| **CKSAAP** | 0.4972 | 0.6222 |
| **KNN** | 0.5611 | 0.6334 |
| **AAindex** | 0.5722 | 0.6415 |
| **SS** | 0.5444 | 0.5916 |
| **seqRSA** | 0.5250 | 0.5772 |
| **Disorder** | 0.5694 | 0.5547 |
| **seqHSE** | 0.5611 | 0.6029 |
| **PSSM** | 0.5778 | 0.6302 |
| **HH** | 0.4972 | 0.6109 |
| **All seq** | 0.5970 | 0.6750 |
| **CHOPS** | 0.6056 | 0.5482 |
| **strHSE** | 0.5361 | 0.5611 |
| **Depth** | 0.5000 | 0.4968 |
| **strRSA** | 0.5333 | 0.5707 |
| **All seq+str** | 0.6310 | 0.6770 |

**Table S10.** Statistical test between the prediction probabilities based on two groups of features for positive and negative samples. *P*-values were calculated by the pairwise one-tailed Wilcoxon signed-rank tests.

| **Residue** | **Classification probability of positive samples** | | | **Classification probability of negative samples** | | |
| --- | --- | --- | --- | --- | --- | --- |
|  | **sequence-based and structure model-based features** | **sequence-based features** | ***P*-value** | **sequence-based and structure model-based features** | **sequence-based features** | ***P*-value** |
| **K** | 0.5528 | 0.5447 | 2.5E-01 | 0.4506 | 0.4801 | 5.0E-04 |
| **R** | 0.5849 | 0.5684 | 1.1E-05 | 0.4088 | 0.4025 | 9.2E-01 |

# **Supporting Figures**


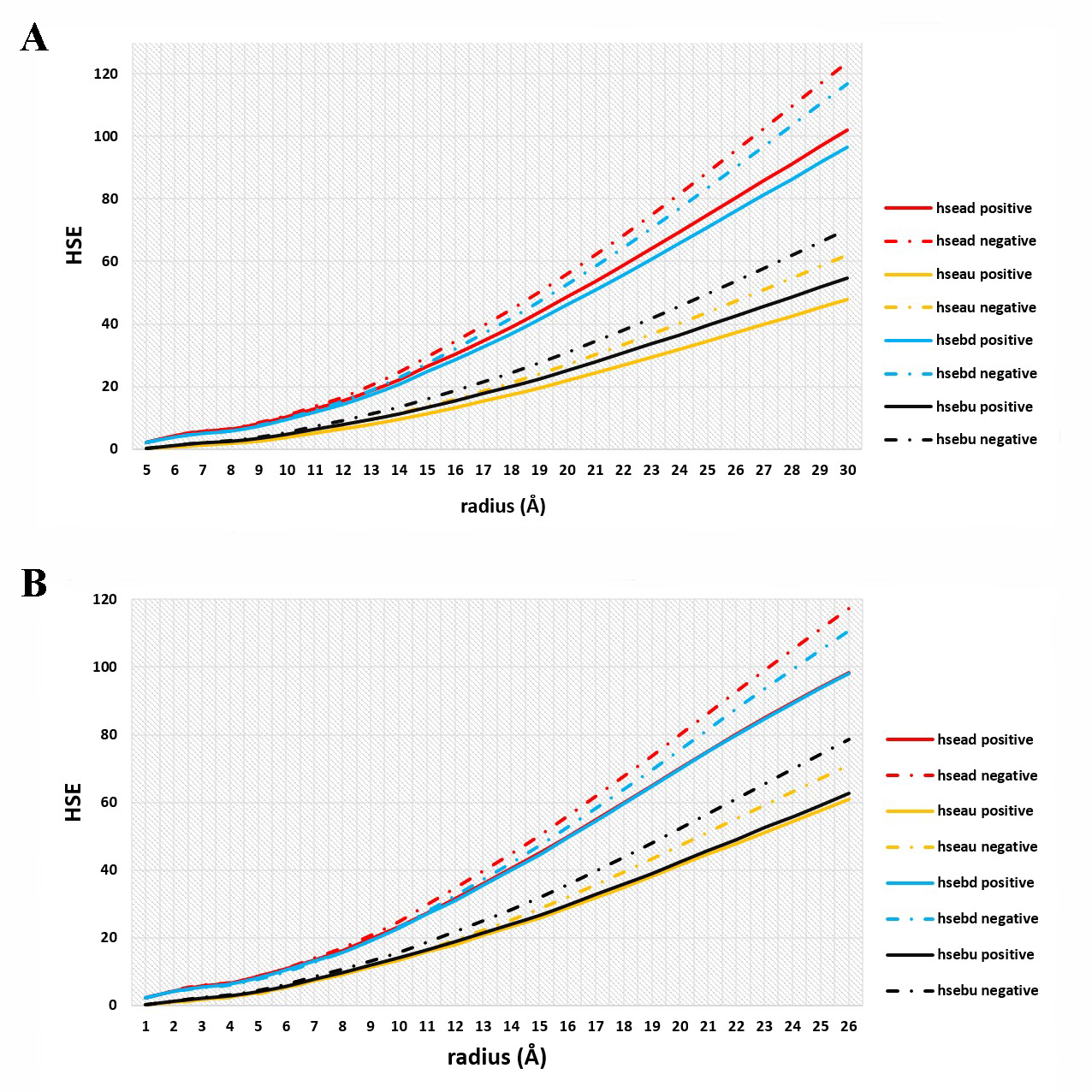


**Figure S1.** (A) Four types of HSE values for methyllysine and non-methyllysine sites with radius from 5Å to 30Å. (B) Four types of HSE values for methylarginine and non-methylarginine sites with radius from 5Å to 30Å.


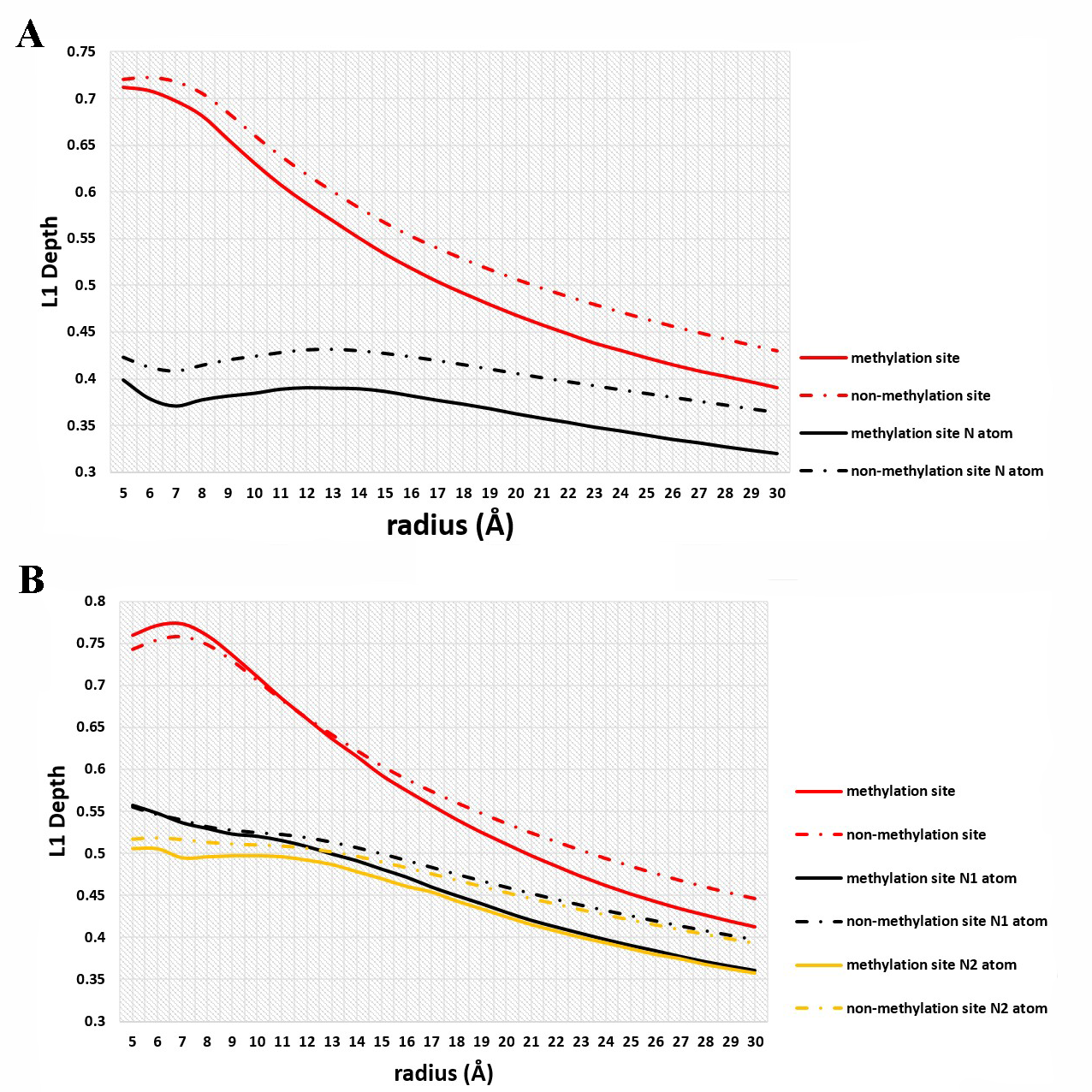


**Figure S2.** (A) The residue-level L_1_ depth and atom-level L_1_ depth for methyllysine and non-methyllysine sites with radius from 5Å to 30Å. (B) The residue-level L_1_ depth and atom-level L_1_ depth for methylarginine and non-methylarginine sites with radius from 5Å to 30Å.

**References**

1 The UniProt, C. UniProt: a worldwide hub of protein knowledge. *Nucleic Acids Research* **47**, D506-D515, doi:10.1093/nar/gky1049 (2018).

2 Berman, H. M. *et al.* The Protein Data Bank. *Nucleic Acids Research* **28**, 235-242, doi:10.1093/nar/28.1.235 (2000).

3 Huang, Y., Niu, B., Gao, Y., Fu, L. & Li, W. CD-HIT Suite: a web server for clustering and comparing biological sequences. *Bioinformatics* **26**, 680-682 (2010).

4 Huang, K.-Y. *et al.* dbPTM in 2019: exploring disease association and cross-talk of post-translational modifications. *Nucleic Acids Research* **47**, D298-D308, doi:10.1093/nar/gky1074 (2018).

5 Ju, Z., Cao, J.-Z. & Gu, H. iLM-2L: A two-level predictor for identifying protein lysine methylation sites and their methylation degrees by incorporating K-gap amino acid pairs into Chou׳s general PseAAC. *Journal of Theoretical Biology* **385**, 50-57, doi:<http://dx.doi.org/10.1016/j.jtbi.2015.07.030> (2015).

6 Lee, T.-Y., Chang, C.-W., Lu, C.-T., Cheng, T.-H. & Chang, T.-H. Identification and characterization of lysine-methylated sites on histones and non-histone proteins. *Computational Biology and Chemistry* **50**, 11-18, doi:https://doi.org/10.1016/j.compbiolchem.2014.01.009 (2014).

7 Deng, W. *et al.* Computational prediction of methylation types of covalently modified lysine and arginine residues in proteins. *Briefings in Bioinformatics* **18**, 647-658, doi:10.1093/bib/bbw041 (2016).

8 Shi, S.-P. *et al.* PLMLA: prediction of lysine methylation and lysine acetylation by combining multiple features. *Molecular BioSystems* **8**, 1520-1527, doi:10.1039/C2MB05502C (2012).

9 Shi, S.-P. *et al.* PMeS: Prediction of Methylation Sites Based on Enhanced Feature Encoding Scheme. *PLoS ONE* **7**, e38772, doi:10.1371/journal.pone.0038772 (2012).

10 Zhang, Z.-H., Wang, Z.-H., Zhang, Z.-R. & Wang, Y.-X. A novel method for apoptosis protein subcellular localization prediction combining encoding based on grouped weight and support vector machine. *FEBS Letters* **580**, 6169-6174, doi:<http://dx.doi.org/10.1016/j.febslet.2006.10.017> (2006).

11 Chen, Z. *et al.* Prediction of Ubiquitination Sites by Using the Composition of k-Spaced Amino Acid Pairs. *PLoS ONE* **6**, e22930, doi:10.1371/journal.pone.0022930 (2011).

12 Chen, X. *et al.* Incorporating key position and amino acid residue features to identify general and species-specific Ubiquitin conjugation sites. *Bioinformatics* **29**, 1614-1622, doi:10.1093/bioinformatics/btt196 (2013).

13 Suo, S.-B. *et al.* Position-Specific Analysis and Prediction for Protein Lysine Acetylation Based on Multiple Features. *PLoS ONE* **7**, e49108, doi:10.1371/journal.pone.0049108 (2012).

14 Henikoff S & JG., H. Amino acid substitution matrices from protein blocks. *Proc. Natl. Acad. Sci. USA* **89**, 10915-10919 (1992).

15 Kawashima, S. *et al.* AAindex: amino acid index database, progress report 2008. *Nucleic Acids Research* **36**, D202-D205, doi:10.1093/nar/gkm998 (2008).

16 Atchey, W. R., Zhao, J., Fernandes, A. D. & Druke, T. Solving the protein sequence metric problem. *Proc. Natl. Acad. Sci. USA* **102**, 6395–6400 (2005).

17 McGuffin, L. J., Bryson, K. & Jones, D. T. The PSIPRED protein structure prediction server. *Bioinformatics* **16**, 404-405, doi:10.1093/bioinformatics/16.4.404 (2000).

18 E. Faraggi, T. Zhang, Y. Yang, Kurgan, L. & Zhou, Y. SPINE X: Improving protein secondary structure prediction by multi-step learning coupled with prediction of solvent accessible surface area and backbone torsion angles. *J. Comput. Chem* **33**, 259-267 (2002).

19 Ward JJ, Sodhi JS, McGuffin LJ, Buxton BF & DT., J. Prediction and functional analysis of native disorder in proteins from the three kingdoms of life. *J Mol Biol.* **337(3)**, 635-645 (2004).

20 Ward, J. J., McGuffin, L. J., Bryson, K., Buxton, B. F. & Jones, D. T. The DISOPRED server for the prediction of protein disorder. *Bioinformatics* **20**, 2138-2139, doi:10.1093/bioinformatics/bth195 (2004).

21 Hamelryck, T. An amino acid has two sides: a new 2D measure provides a different view of solvent exposure. *Proteins* **59(1)**, 38-48 (2005).

22 Sweredoski, M. J. & Baldi, P. PEPITO: improved discontinuous B-cell epitope prediction using multiple distance thresholds and half sphere exposure. *Bioinformatics* **24**, 1459-1460, doi:10.1093/bioinformatics/btn199 (2008).

23 Zheng, W. *et al.* Analysis of Conformational B-Cell Epitopes in the Antibody-Antigen Complex Using the Depth Function and the Convex Hull. *PLoS ONE* **10**, e0134835, doi:10.1371/journal.pone.0134835 (2015).

24 Heffernan, R. *et al.* Highly accurate sequence-based prediction of half-sphere exposures of amino acid residues in proteins. *Bioinformatics (Oxford, England)* (2015).

25 S F Altschul *et al.* Gapped BLAST and PSI-BLAST: a new generation of protein database search programs. *Nucleic Acids Res* **25(17)**, 3389–3402 (1997).

26 Remmert, M., Biegert, A., Hauser, A. & Soding, J. HHblits: lightning-fast iterative protein sequence searching by HMM-HMM alignment. *Nat Meth* **9**, 173-175, doi:<http://www.nature.com/nmeth/journal/v9/n2/abs/nmeth.1818.html#supplementary-information> (2012).

27 Hubbard, S. J. & Thornton, J. M. *NACCESS*. (1993).

28 Chakravarty, S. & Varadarajan, R. Residue depth: a novel parameter for the analysis of protein structure and stability. *Structure* **7**, 723-732, doi:<http://dx.doi.org/10.1016/S0969-2126(99)80097-5> (1999).

29 Vardi, Y. & Zhang, C.-H. The multivariate L1-median and associated data depth. *Proceedings of the National Academy of Sciences* **97**, 1423-1426 (2000).
